# The spatiotemporal neural dynamics of object location representations in the human brain

**DOI:** 10.1038/s41562-022-01302-0

**Published:** 2022-02-24

**Authors:** Monika Graumann, Caterina Ciuffi, Kshitij Dwivedi, Gemma Roig, Radoslaw M. Cichy

**Affiliations:** 1 Department of Education and Psychology, Freie Universität Berlin, Berlin, Germany; 2 Berlin School of Mind and Brain, Faculty of Philosophy, Humboldt-Universität zu Berlin, Berlin, Germany; 3 Department of Computer Science, Goethe Universität, Frankfurt am Main, Germany; 4 Bernstein Center for Computational Neuroscience Berlin, Berlin, Germany

**Keywords:** Perception, Neural decoding, Learning algorithms, Extrastriate cortex, Object vision

## Abstract

To interact with objects in complex environments, we must know what they are and where they are in spite of challenging viewing conditions. Here, we investigated where, how and when representations of object location and category emerge in the human brain when objects appear on cluttered natural scene images using a combination of functional magnetic resonance imaging, electroencephalography and computational models. We found location representations to emerge along the ventral visual stream towards lateral occipital complex, mirrored by gradual emergence in deep neural networks. Time-resolved analysis suggested that computing object location representations involves recurrent processing in high-level visual cortex. Object category representations also emerged gradually along the ventral visual stream, with evidence for recurrent computations. These results resolve the spatiotemporal dynamics of the ventral visual stream that give rise to representations of where and what objects are present in a scene under challenging viewing conditions.

## Abstract (web summary)

Using electroencephalography and functional magnetic resonance imaging, Graumann et al. examine where, how and when representations of object location and category emerge in the human brain.

## Main

To interact with objects in our environments, the two arguably most basic questions that our brains must answer are what objects are present and where they are. To address the first question and identify an object, we must recognize objects independently of the viewing conditions of a given scene, such as where the object is located. A large body of research has shown that the ventral visual stream^1–4^, a hierarchically interconnected set of regions, achieves this by transforming retinal input in successive stages marked by increasing tolerance and complexity. At its high stages in high-level ventral visual cortex, object representations are tolerant to changes in retinotopic location^5–7^.

In contrast, we know considerably less about how the brain determines where an object is located. Current empirical data imply three different theoretical accounts.

**Fig. 1:**
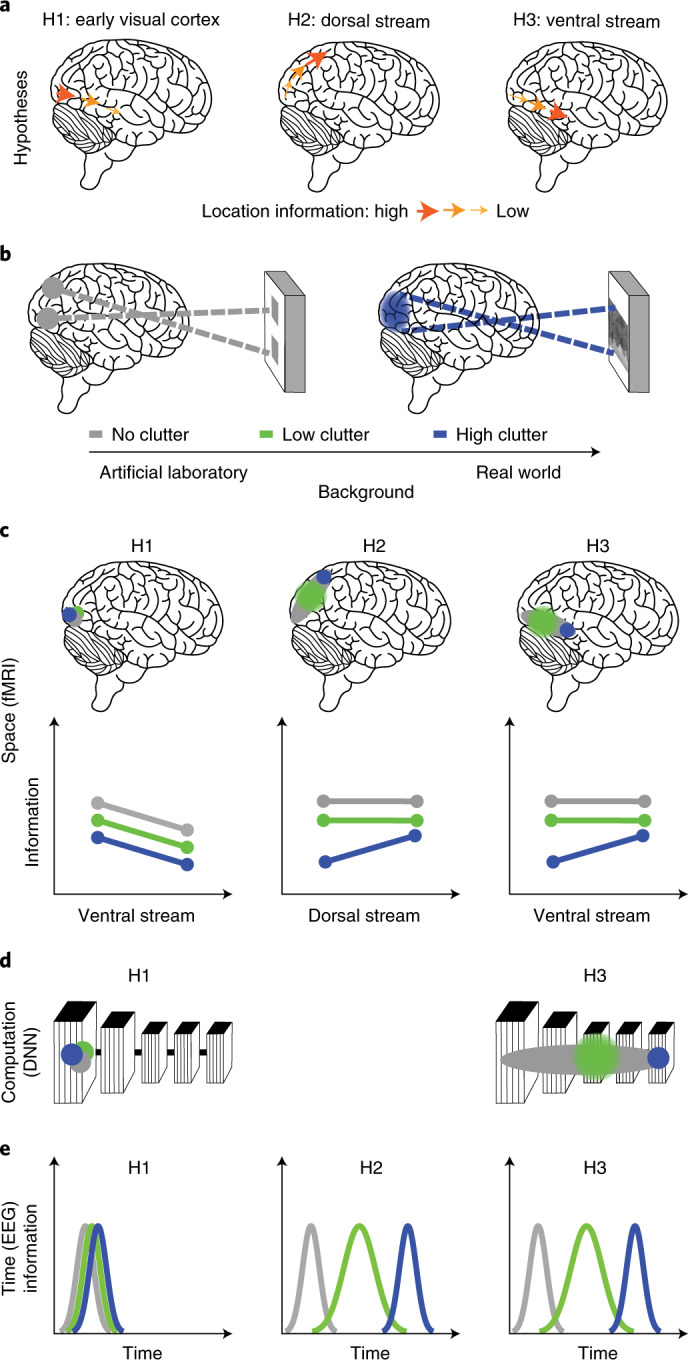
Hypotheses and predictions about the pathway of object location representations in the human brain. **a**, H1: representations of object location emerge in early visual cortex and degrade along further processing stages. H2 and H3: object location representations emerge gradually along the dorsal (H2) or ventral (H3) visual stream. **b**, Left: when objects are presented on a blank background, object location in the visual field maps retinotopically onto early visual cortex, allowing for direct location read-out (grey). Right: when objects appear in a cluttered scene, large parts of early visual cortex are activated, hindering a direct read-out (blue). Representations are quantified as linearly classifiable object location information from brain or model activity patterns^1^. **c**, Predictions in space, colour-coded by background condition: no (grey), low (green) and high (blue) clutter. H1 predicts that independent of the object’s background, location information for the object is highest in early processing stages in space. H2 and H3 predict similar levels of location information with no clutter across the entire processing pathway in all assessments. For highly cluttered backgrounds, H2 and H3 predict the emergence of location representations in late processing stages of the dorsal (H2, **c**) and ventral (H3, **c**) stream. Location information in the low-clutter condition is expected to be in between the no- and the high-clutter condition. **d**, Computational model of the ventral visual stream. H1 (left) predicts highest location information in early layers of the model in all conditions. H3 (right) predicts high location information across all layers with no clutter and highest location information in late layers with high clutter. Location information in the low-clutter condition is expected to be in between the other two conditions. Since this is a model of the ventral stream, it does not make predictions about the dorsal stream (H2). **e**, Location information in time. H1 predicts that location information peaks early in time in all conditions. Both H2 and H3 predict an early peak with no and a late peak with high clutter. The peak for low clutter is expected to be in between no and high clutter.

One hypothesis (H1) is that object location representations are already present at the early stages of visual processing (H1, Fig. 1a) and thus no further computation is required. Given the idea that ventral stream representations become successively more tolerant to changes in viewing conditions such as location^1^, it seems plausible that object location representations are to be found at the early stages of the processing hierarchy. Consistent with this view, human studies using multivariate analysis have shown that object location is often strongest in early visual cortex^8,9^, likely related to its small receptive field size which allows for spatial coding with high resolution^10^.

An alternative account (H2) is that location representations emerge in the dorsal visual stream (H2, Fig. 1a)^11^. This view is supported by findings from neuropsychology^2,4,11^ and by studies finding object location information along the dorsal pathway^2,12^.

A third possibility is that location representations emerge through extensive processing but in the ventral visual stream (H3, Fig. 1a). This view receives support from the observation that object location information was found across the entire ventral visual stream including high-level ventral visual cortex in human^5,8,9,13^ and non-human primates^14^. In line with these observations, high-level ventral visual cortex is known to be retinotopically organized^15–17^ and exhibits an eccentricity bias^18–20^.

How can we adjudicate between these hypotheses given the mixed empirical support? We propose that it is key to acknowledge the importance of assessing object location representations under conditions that increase the complexity of the visual scene to increase ecological validity. Previous research typically investigated object location representations by presenting cut-out objects on blank backgrounds. This creates a direct mapping between the location of visual stimulation and the active portions of retinotopically organized cortex (Fig. 1b, left). In contrast, in daily life, objects appear on backgrounds cluttered by other elements^21,22^. This activates a large swath of cortex, independently of where the object is (Fig. 1b, right). Whereas in the former case location information can be directly accessible through retinotopic activation in early visual areas (supporting H1), in the latter case additional processing might be required to distil out location information (supporting H2 or H3).

Taking the importance of background into consideration, we used a combination of methods to distinguish between the proposed theoretical hypotheses. We used functional MRI (fMRI), deep neural networks (DNNs) and electroencephalography (EEG) to assess where, how and when location representations emerge in the human brain. We quantified the presence of location representations by the performance of a multivariate pattern classifier to predict object location from brain measurements.

Assessed in this way, the predictions for the hypotheses are as follows: If H1 is correct, independent of the nature of the object’s background, object location information peaks in early visual cortex (Fig. 1c, left), early in the DNN processing hierarchy (Fig. 1d, left) and early during visual processing (Fig. 1e, left). For H2 and H3, the prediction of peak location information depends on the background. For cut-out isolated objects, location information is high across the entire dorsal and ventral pathways, and the processing hierarchy of the DNN (Fig. 1c,d, middle and right, grey). In contrast, for objects appearing on cluttered backgrounds, object location information emerges late in the DNN hierarchy (Fig. 1d, right, blue) and late in time (Fig. 1e, middle and right, blue). H2 and H3 differ in predicting location information to peak in dorsal (Fig. 1c, middle, blue) or ventral visual cortex (Fig. 1c, right, blue), respectively.

To anticipate, our results strongly support H3. When objects appear on cluttered backgrounds, object location representations emerge late in the hierarchy of the ventral visual stream and of the DNN, as well as late in time, indicating recurrent processing. A corresponding analysis of object category representations revealed an equivalent pattern of results with emergence along the ventral visual stream and temporal dynamics suggesting recurrence. Taken together, our results resolve where, when and how object representations emerge in the human brain when objects are viewed under more challenging viewing conditions.

## Results

**Fig. 2:**
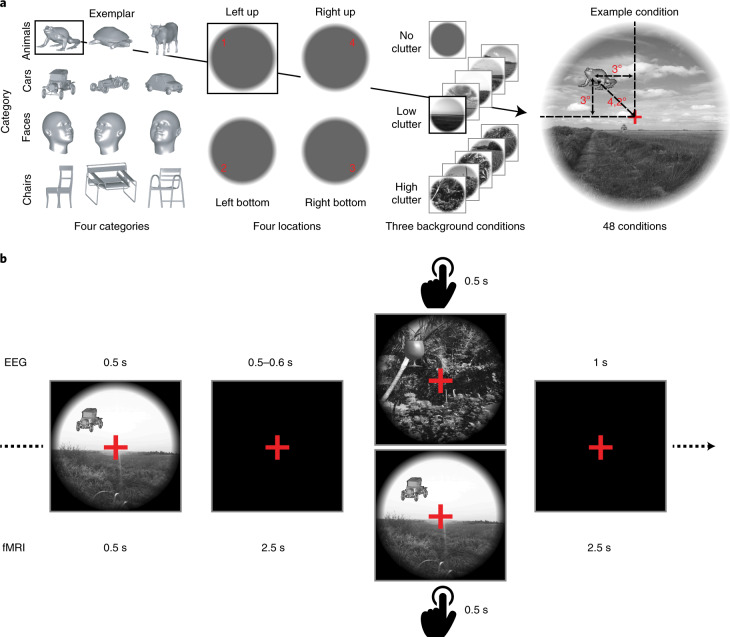
Experimental design and tasks. **a**, Experimental design. We used a fully crossed design with factors of object category, location and background. Note that, for copyright reasons, all example backgrounds shown are for illustrative purposes and were not used in the experiment. **b**, Tasks. The experimental design was adapted to the specifics of each modality by adjusting the interstimulus interval. On each trial, participants viewed images for 500 ms followed by a blank interval (0.5–0.6 s in EEG, 2.5 s in fMRI). The task was to respond with button press to catch trials that were presented on every fourth trial on average. Catch trials were marked by the presence of a probe (glass) in the EEG experiment and by an image repetition (one-back) in the fMRI experiment. Image presentation was followed by blank screen (1 s in EEG, 2.5 s in fMRI).

To investigate where, how and when representations of object location emerge in the brain, we created a visual stimulus set (Fig. 2a) with the three orthogonal factors objects (three exemplars each in four object categories), locations (four quadrants) and backgrounds (three kinds: uniform grey, low- and high-cluttered natural scenes, referred to as ‘no’, ‘low’ and ‘high’ clutter). Collapsing across exemplars, we used a fully crossed design with four categories × four locations × three background conditions, resulting in 48 stimulus conditions. This design allowed us to also investigate representations of object category as a secondary question of the study.

To resolve human brain responses with high spatial and temporal resolution, participants viewed images from the stimulus set while we recorded fMRI (*N* = 25) and EEG (*N* = 27) data in separate sessions. Experimental parameters were optimized for each imaging modality (Fig. 2b). On each trial, participants viewed individual stimuli while fixating on a central fixation cross and performing a one-back (fMRI) or a detection task (EEG) to direct participants’ attention towards the images (Fig. 2b). Response trials were excluded from analysis.

**Fig. 3:**
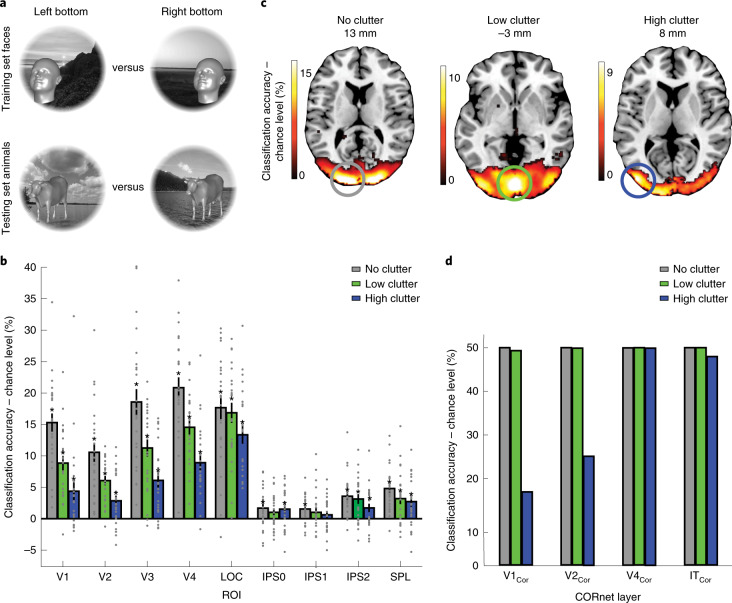
fMRI results of location classification. **a**, Classification scheme for object location across category. We trained an SVM to distinguish between brain activation patterns evoked by objects of a particular category presented at two locations (here: faces bottom left and right) and tested the SVM on activation patterns evoked by objects of another category (here: animals) presented at the same locations. Objects are enlarged for visibility and did not extend into another quadrant in the original stimuli. **b**, Location classification in early visual cortex, ventral and dorsal visual ROIs (*N* = 25, two-tailed Wilcoxon signed-rank test, *P* < 0.05, FDR corrected). With no clutter, location information was high across early visual cortex and ventral ROIs. In the low- and high-clutter conditions, location representations emerged gradually along the ventral stream. In dorsal ROIs, location information was low, independent of background condition. Stars above bars indicate significance above chance (see Supplementary Tables 1, 2 and 3 for *P* values). Error bars represent *s.e.m*. Dots represent single subject data. **c**, fMRI searchlight result for classification of object location (*N* = 25, two-tailed Wilcoxon signed-rank test, *P* < 0.05, FDR corrected). Peak classification accuracy is indicated by colour-coded circles (no clutter: left V3 (grey, *XYZ* coordinates −19 mm, −97 mm, 13 mm); low clutter: left V1 (green, −5 mm, −86 mm, −3 mm); high clutter: left LOC (blue, −44 mm, −83 mm, 8 mm)). Millimetres (mm) indicate axial slice position along *z* axis in Montreal Neurological Institute space. **d**, Location classification in a DNN. In the high-clutter condition, location information emerged along the processing hierarchy, analogous to the ventral visual stream.

We used multivariate pattern classification to track the emergence of object location representations. We consider the peaks in information, that is, in classification, as indicators of where (fMRI) and when (EEG) location representations become most untangled and are thus explicitly represented^1^. In each case, we trained a support vector machine (SVM) to pairwise classify between activation patterns belonging to one object category shown at two different locations (Fig. 3a, faces at bottom left and right). We then tested the SVM on activation patterns of the same locations with a new object category (Fig. 3a, animals at bottom left and right). Repeated for all combinations of locations and categories, the averaged classification accuracy quantifies object location information independent of object category. This procedure was performed in a space-resolved fashion for fMRI and in a time-resolved fashion for EEG (see Supplementary Fig. 1a for details).

### The locus of object location representations

To determine the locus of object location representations, we used a regions of interest (ROI) fMRI analysis, including early visual regions (V1, V2 and V3) shared to the hierarchy of the ventral (V4 and LOC^23^) and the dorsal visual stream (intraparietal sulcus: IPS0, IPS1, IPS2 and superior parietal lobule (SPL)).

As expected, we found that most regions contained above-chance level location information in all background clutter conditions (Fig. 3b; *N* = 25, two-tailed Wilcoxon signed-rank test, *P* < 0.05, FDR corrected; see Supplementary Table 1 for *P* values). However, the amount of location information depended critically on the brain region and background condition.

Focusing on the ventral visual stream first, we observed similar amounts of location information across regions when objects were presented without clutter (Fig. 3b, grey bars). In contrast, when objects were presented on cluttered backgrounds, location information emerged along the ventral visual processing hierarchy with less information in early visual areas than in LOC (Fig. 3b, green and blue bars; *N* = 25, 5 × 3 repeated-measures ANOVA, post hoc *t* tests Tukey corrected; see Supplementary Table 2 for *P* values). These results are at odds with H1, which predicts that location information decreases along the ventral stream independent of background condition. Instead, the observed increase of location information along the ventral visual stream with cluttered backgrounds is consistent with H3.

**Fig. 4:**
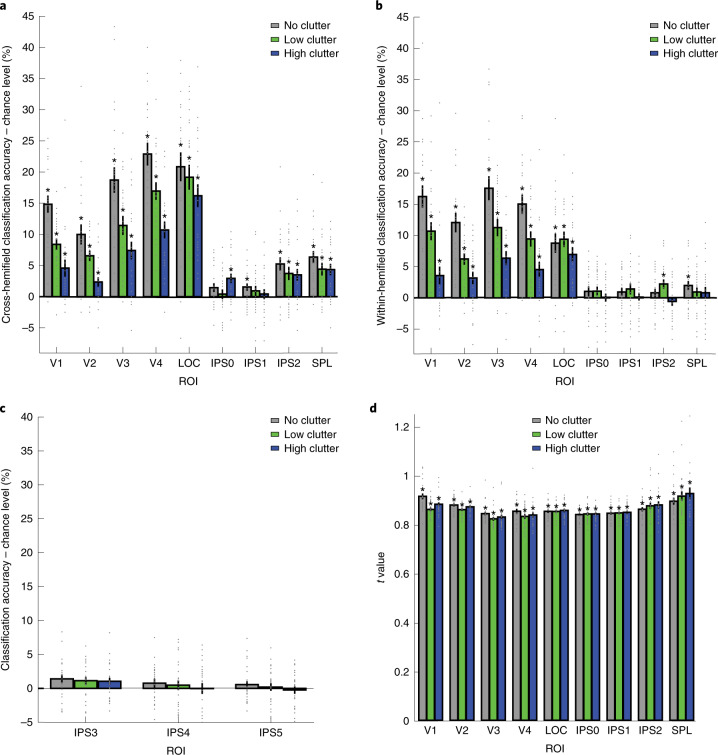
Location classification within and across hemifields, in IPS3–5 and univariate ROI results. **a**, Results of location classification across categories between visual hemifields (left up versus right up, left bottom versus right bottom). Similar to the classification across four locations, the repeated-measures ANOVA along the ventral stream (five ROIs × three clutter levels) yielded significant main (ROI: *F*_(4,96)_ = 24.62, *P* < 0.001, partial *η*^2^ = 0.51; background: *F*_(1.49,35.85)_ = 45.34, *P* < 0.001, partial *η*^2^ = 0.65) and interaction effects (*F*_(8,192)_ = 2.95, *P* = 0.004, partial *η*^2^ = 0.11). Post hoc tests yielded results comparable to the main results (V1, V2 and V3 < LOC with high clutter). Stars above bars indicate significance above chance (*N* = 25, two-tailed Wilcoxon signed-rank test, *P* < 0.05, FDR corrected). **b**, Location classification across categories within visual hemifields (left up versus left bottom, right up versus right bottom). As for the main analysis, the ANOVA yielded significant main (ROI: *F*_(4,96)_ = 4.16, *P* = 0.004, partial *η*^2^ = 0.15; background: *F*_(1.60,38.43*)*_ = 57.90, *P* < 0.001, partial *η*^2^ = 0.71) and interaction effects (*F*_(8,192)_ *=* 5.84, *P* < 0.001, partial *η*^2^ = 0.20). The post hoc tests revealed a significant difference between V3 and LOC in the noclutter condition (*P* = 0.030). Stars above bars indicate significance above chance (*N* = 25, two-tailed Wilcoxon signed-rank test, *P* < 0.05, FDR corrected). **c**, Classification accuracies in IPS3, IPS4 and IPS5 were not significantly higher than chance level in all background conditions (*N* = 25, two-sided Wilcoxon signed-rank test, *P* > 0.05, FDR corrected). Error bars represent *s.e.m*. Dots represent single/subject data. **d**, Absolute *t* values in each background condition and ROI, averaged across locations and categories. A 9 × 3 repeated-measures ANOVA with factors ROI and clutter revealed a significant main effect of ROI (*F*_(2.60,62.43)_ = 9.19, *P* < 0.001, partial *η*^2^ = 0.18) and a significant interaction effect (*F*_(3.40,81.64)_ = 9.89, *P* < 0.001, partial *η*^2^ = 0.03). Significant post hoc tests are listed in Supplementary Table 4. Overall, post hoc tests showed no clear pattern of results between early, ventral and dorsal areas, except for higher activation in V1 than in dorsal areas and LOC with no clutter. Stars above bars indicate significance above chance (*N* = 25, two-tailed Wilcoxon signed-rank test, *P* < 0.05, FDR corrected).

We ascertained these observations statistically with a 5 × 3 repeated-measures ANOVA with factors ROI (V1, V2, V3, V4 and LOC) and background (no, low and high clutter). Besides both main effects (ROI: *F*_(4,96)_ = 18.30, *P* < 0.001, partial *η*^2^ = 0.43; background: *F*_(1.44,34.48)_ = 64.11, *P* < 0.001, partial *η*^2^ = 0.73), we crucially found the interaction to be significant (*F*_(8,192)_ = 5.40, *P* < 0.001, partial *η*^2^ = 0.18). As the interaction makes the main effects difficult to interpret, we conducted post hoc paired *t* tests (all reported in Supplementary Table 2, Tukey corrected). The statistical analysis confirmed all the qualitative observations: There were no significant differences between ROIs in the no-clutter condition, except between V2 and V3 (*P* = 0.009) and between V2 and V4 (*P* = 0.001). There was more location information in LOC than in V1, V2 and V3 when background clutter (both low and high) was present than when it was not (Fig. 3b; all *P* < 0.03, see Supplementary Table 2 for *P* values, Tukey corrected). This effect was robust for the comparison of locations across, but not within, visual hemifields (Fig. 4a,b): post hoc tests comparing early visual areas versus LOC in the high-clutter condition were significant for the cross-hemifield classification (Fig. 4a; V1: *P* = 0.003; V2: *P* < 0.001; V3: *P* = 0.004, Tukey corrected), but not for the within-hemifield classification (Fig. 4b; V1: *P* = 0.697; V2: *P* = 0.281; V3: *P* = 1.00, Tukey corrected).

Focusing next on the dorsal visual stream, we observed low object location information independent of background condition (Fig. 3b; *N* = 25, 7 × 3 repeated-measures ANOVA). In the no- and low-clutter conditions, location information was higher in early visual cortex than in dorsal regions (*N* = 25, post hoc *t* tests, Tukey corrected; see Supplementary Table 3 for *P* values). This is inconsistent with H2, which predicts an increase of object location information along the dorsal stream.

Consistent with these qualitative observations, statistical testing by 7 × 3 repeated-measures ANOVA with factors ROI (V1, V2, V3, IPS0, IPS1, IPS2 and SPL) and background (no, low and high clutter) did not provide statistical evidence for H2. We found significant main (ROI: *F*_(3.16,75.93)_ = 36.2, *P* < 0.001, partial *η*^2^ = 0.60; background: *F*_(2,48)_ = 35.8, *P* < 0.001, partial *η*^2^ = 0.60) and interaction effects (*F*_(6.25,149.89)_ = 14.5, *P* < 0.001, partial *η*^2^ = 0.38). The post hoc tests showed that location information was higher in V1, V2 and V3 compared with dorsal regions in the no- and low-clutter conditions (Fig. 3b, grey and green, except V1 and V2 versus IPS2 and SPL with low clutter, which were n.s.; see Supplementary Table 3 for *P* values). With high clutter, there was more location information in V3 than in IPS0, IPS1 and IPS2. Location classification in IPS3, IPS4 and IPS5 did not reveal significant information above chance level (Fig. 4c; *N* = 25, two-tailed Wilcoxon signed-rank test, all *P* > 0.05 FDR corrected, see Supplementary Table 1 for *P* values). Univariate responses were comparable across regions overall (Fig. 4d). Post hoc tests to a 9 × 3 repeated-measures ANOVA with factors ROI (V1, V2, V3, V4, LOC, IPS0, IPS1, IPS2 and SPL) and background (no, low and high clutter) revealed that responses were significantly higher in V1 compared with the other ROIs in the no-clutter condition (all *P* < 0.03; all *P* values listed in Supplementary Table 4, Tukey corrected), but there was no significant difference in activation between LOC and dorsal areas (Fig. 4d, all *P* values in Supplementary Table 4, Tukey corrected).

To explore whether any other brain regions beyond the investigated ROIs contain location information, we used a spatially unbiased fMRI searchlight analysis^24^. We did not find statistical evidence for location information beyond the ventral and dorsal stream, and the pattern of results was consistent with the outcome of the ROI analysis (Supplementary Fig. 2). There was widespread location information (*N* = 25, two-tailed Wilcoxon signed-rank test, *P* < 0.05, FDR corrected) from the occipital cortex up into the dorsal (precuneus, superior parietal lobule) and ventral (fusiform gyrus) visual stream. Depending on background condition, location information peaked in different visual areas. In the no-clutter condition, the peak was in left V3, in the low-clutter condition in left V1 and in the high-clutter condition in left LOC (Fig. 3c, see caption for coordinates). Distances between peaks were significantly larger than chance (*N* = 25, bootstrapping of condition labels, 10,000 bootstraps, *P* < 0.05 one-tailed bootstrap test against chance level, Bonferroni corrected) between the no- and the high-clutter condition (Euclidean distance 15.9, CI 1.0–3.6, *P* < 0.001) and between the low- and the high-clutter condition (Euclidean distance 22.0, CI 2.0–16.3, *P* = 0.002), but not for the no- and low-clutter condition (Euclidean distance 13.6, CI 1.4–14.7, *P* = 0.275).

Together, these results provide consistent evidence for the hypothesis that representations of object location across visual hemifields emerge in the ventral visual stream (H3) when objects appear in cluttered scenes.

### Computational modelling

DNNs trained on object categorization are currently the best predicting models of ventral visual stream representations^25–27^ and show a spatiotemporal correspondence in their processing hierarchy to the visual brain^25,28–30^. Therefore, they constitute feasible biologically inspired models for computing complex visual representations^28,31^. If such DNNs are useful models of visual processing in human visual cortex, they should show a similar pattern of results as the ventral visual stream in the representation of object location, too.

To evaluate this prediction, we chose the recurrent CORnet-S model because it is among the best-performing models on a benchmark for predicting neural responses in monkey inferior temporal cortex (IT)^26,27^ and approximates explicitly the hierarchy of the ventral visual system. Each region of the ventral stream is modelled as one processing block with a corresponding name (V1_Cor_, V2_Cor_, etc.). Analogous to the fMRI analysis, we extracted the unit activation patterns to our stimulus set at the last layer of each block and classified object location across category to identify the processing stage of the DNN at which object location representations emerge (Fig. 3d).

We found that in the no- and low-clutter conditions, location information was at or close to ceiling in all layers. In the high-clutter condition however, location information was low in V1_Cor_ and emerged along the processing hierarchy. Qualitatively equivalent results were obtained in three other DNNs (Alexnet, ResNet-50 and CORnet-Z; Supplementary Fig. 3a–c), demonstrating the generalizability of the results pattern. This result was still robust in all four DNNs when limiting the classification to either horizontal or vertical location comparisons (Supplementary Fig. 3e,f).

In sum, we found that DNNs trained on object categorization show a similar pattern of location representations along their processing hierarchy as the human brain. This demonstrates how object location representations might be computed in biological systems. This result lends independent evidence against H1 and yields plausibility to H3 since CORnet-S was built to model the ventral stream. However, this result cannot disambiguate between H2 and H3, as models of this kind have been found to predict human brain activity in both the ventral and dorsal stream^32,33^.

### Temporal dynamics of object location representations

We conducted time-resolved multivariate EEG analysis to determine the time course with which object location representations emerge. The general analysis scheme was the same as for the fMRI analysis presented above (Fig. 3a) but applied to time-specific EEG channel activation patterns rather than fMRI activation patterns.

**Fig. 5:**
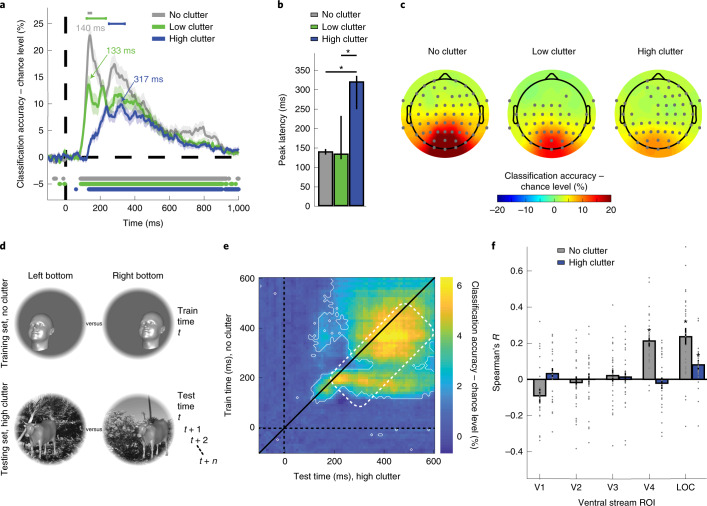
Temporal dynamics of object location representations. **a**, Results of time-resolved location classification across category from EEG data. Results are colour coded by background condition, with significant time points indicated by lines below curves (*N* = 27, two-tailed Wilcoxon signed-rank test, *P* < 0.05, FDR corrected), 95% CI of peak latencies indicated by lines above curves. Shaded areas around curves indicate *s.e.m*. Inset text at arrows indicates peak latency (140 ms, 133 ms and 317 ms in the no-, low- and high-clutter condition, respectively). **b**, Comparison of peak latencies of curves in **a**. Error bars represent 95% CI. Stars indicate significant peak latency differences (*P* < 0.05; *N* = 27, bootstrap test with 10,000 bootstraps). **c**, Results of location across category classification searchlight in EEG channel space at peak latencies in no-, low- and high-clutter condition, down-sampled to 10 ms steps. Significant electrodes are marked in grey (*N* = 27, two-tailed Wilcoxon signed-rank test, *P* < 0.05, FDR corrected across electrodes and time points). **d**, Time generalization analysis scheme for classifying object location across category and background condition. The classification scheme was the same as in **a** with the differences that (i) the training set conditions always came from the no-clutter while the testing set conditions came from the high-clutter condition and (ii) training and testing was repeated across all combinations of time points for a peri-stimulus time window between −100 and 600 ms (see Supplementary Fig. 1b for details). Objects are enlarged for visibility and did not extend into another quadrant in the original stimuli. **e**, Results of the time generalization analysis. Dashed black lines indicate stimulus onset; oblique black line highlights the diagonal. Solid white outlines indicate significant time points (*N* = 27, two-tailed Wilcoxon signed-rank test, *P* < 0.05, FDR corrected). Dashed white outline highlights delayed clusters. **f**, EEG–fMRI fusion. Results represent the correlations between single-subject fMRI RDVs of classification accuracies and group-averaged RDVs of the EEG peaks in **a**. Stars above bars indicate significance above chance (*N* = 27, two-tailed Wilcoxon signed-rank test, *P* < 0.05, FDR corrected). Error bars represent the *s.e.m*. Dots represent single-subject data points.

The analysis revealed location information for all background clutter levels (Fig. 5a, *N* = 27, two-tailed Wilcoxon signed-rank test, *P* < 0.05, FDR corrected), but with different dynamics (Fig. 5b, see Supplementary Table 5 for classification onsets and peak values). We report peak latencies with 95% confidence intervals (*N* = 27, 10,000 bootstraps). Whereas the peak latency was similar for the no- (140 ms (133–147 ms)) and the low-clutter (133 ms (121–233 ms)) condition, it was delayed in the high-clutter condition (317 ms (250–336 ms)). Statistical analysis (*N* = 27, bootstrap test, 10,000 bootstraps, *P* < 0.05, one-tailed bootstrap test against zero, FDR corrected) ascertained that the peak latency difference was significant between the high-clutter and the no-clutter conditions (*N* = 27, 177 ms (94–190 ms), *P* < 0.001) and between the high- and the low-clutter conditions (184 ms (16–196 ms), *P* = 0.023), but not between the no- and the low-clutter conditions (7 ms (−11–156 ms), *P* = 0.620). These delays were also robust when classifying locations across or within visual hemifields (Supplementary Fig. 4). A searchlight in EEG sensor space showed that location information at the peaks of the three background conditions was highest at occipital, occipito-parietal and occpito-temporal electrodes (Fig. 5c; *N* = 27, two-tailed Wilcoxon signed-rank test, *P* < 0.05, FDR corrected across electrodes and time points; see Supplementary Fig. 5a–c for time courses), suggesting sources in those areas, which is in line with the fMRI searchlight results (Supplementary Fig. 2) and with univariate EEG topographies (Supplementary Fig. 5d–f).

In sum, this result shows that object location representations emerge later when objects appear on cluttered backgrounds than when they appear on blank backgrounds. This provides further concurrent evidence against H1 and is consistent with H2 and H3, that is, that object location representations emerge at late stages of visual processing when objects are viewed under complex visual conditions.

How is the delay in the peak latencies of the no- and the high-clutter conditions to be interpreted? Assuming that in object processing the brain runs through a series of distinct stages, we see two possible explanations.

One explanation is that the peak latency delay indicates a change in the processing stage at which object location representations emerge. This would mean that in the no-clutter condition, location representations emerge in an early stage whereas with high clutter they emerge during a different, later processing stage (the ‘change’ hypothesis). An alternative explanation is that the processing stage at which object location representations emerge remains the same, but its emergence is delayed in time in the high-clutter condition (the ‘delay’ hypothesis).

To distinguish between these explanations, we used temporal generalization analysis^34^, comparing the representational dynamics with which object location representations emerge in the no- and the high-clutter conditions across time (Fig. 5d). Used in this way, the time generalization analysis yields a two-dimensional matrix indexed in time, indicating at which time points location representations in the no- and the high-clutter conditions are similar. We implemented time generalization by classifying object location across category and background condition for all time point combinations (Fig. 5d and Supplementary Fig. 1b). Overall, we observed a large significant cluster of above-chance classification accuracies across the time generalization matrix (*N* = 27, two-tailed Wilcoxon signed-rank test, *P* < 0.05, FDR corrected). While the ‘change’ hypothesis predicts highest classification accuracies on the diagonal, the ‘delay’ hypothesis predicts highest classification accuracies below the diagonal. The results are reported in Fig. 5e. We found that peak latencies in location information as tested across subjects were significantly shifted below the diagonal (mean Euclidean distance 56.31 ms; *N* = 27, two-tailed Wilcoxon signed-rank test, *P* < 0.001, *r* = 0.65, *s.e.m.* 1.55; see Supplementary Fig. 6a for single-subject peaks), indicating that location representations in the no-clutter condition generalized to the high-clutter condition at later time points (Fig. 5e, white dashed outline). This result was confirmed in a supplementary analysis on the group-averaged peak in Fig. 5e (Euclidean distance 49.50 ms; 10,000 bootstraps; one-tailed bootstrap test against zero, *P* = 0.010; 95% CI 14.14–77.78). Classification accuracies were significantly higher below than above the diagonal between ~120 and 240 ms in the no-clutter condition and from ~200 ms in the high-clutter condition (*N* = 27, two-tailed Wilcoxon signed-rank test, *P* < 0.05, FDR corrected; Supplementary Fig. 6b).

Together, these results provide evidence for the ‘delay’ hypothesis and demonstrate that object location representations in the no- and the high-clutter condition emerge at the same processing stage with a temporal delay.

### Spatiotemporal similarity of location representations

Temporal delays for the same processing stage cannot be explained by a purely feedforward process, suggesting instead the involvement of recurrent processing. Recurrent processes could account for the observed delay with lateral connections within the same area^35,36^. The shared processing stage underlying early and late location representations in the no- and the high-clutter conditions should have a common origin in space, too. Based on the fMRI results, we hypothesized that this origin would be in LOC. To test this hypothesis directly, we used EEG–fMRI fusion based on representational similarity of object location representations^37–39^.

The processing stage at which location representations emerge corresponds to the peak latency of location classification in the EEG for the no- and the high-clutter condition. We thus determined whether location representations identified with EEG at these time points are representationally similar to those identified with fMRI in ventral stream regions for the no- and the high-clutter condition separately. Specifically, we averaged the representational dissimilarity vectors (RDVs) of the time-resolved EEG classification accuracies in Fig. 5a across subjects and time points within the 95% confidence intervals over the peaks. This yielded one RDV per background condition that was then correlated with the single-subject RDV of an fMRI ROI in the same background condition. Results within background and ROI were averaged across fMRI participants.

We found a spatiotemporal correspondence with EEG peak latency for the no-clutter condition in V4 and LOC but for the high-clutter condition in LOC only (Fig. 5f; *N* = 25, two-tailed Wilcoxon signed-rank test, *P* < 0.05, FDR corrected). This establishes LOC as the cortical locus at which object location representations emerge independent of background condition, but involving additional recurrent processing when the background is cluttered. Post hoc tests to a 5 × 2 repeated-measures ANOVA with factors ROI (V1, V2, V3, V4 and LOC) and clutter (no and high) additionally showed that correlations were higher in V4 and LOC than in V1, V2 and V3 with no clutter (see Supplementary Table 6 for *P* values; main effect of ROI: *F*_(4,96)_ = 14.30, *P* < 0.001, partial *η*^2^ = 0.37; n.s. main effect of background: *F*_(1,24)_ = 3.62, *P* = 0.069; interaction: *F*_(4,96)_ = 8.17, *P* < 0.001, partial *η*^2^ = 0.25). The notion that location representations emerge in LOC with recurrence when background is cluttered finds further support from a supplementary analysis showing that location representations with no and high clutter were significantly similar in LOC, but not in other regions (Supplementary Fig. 7; *N* = 25, two-tailed Wilcoxon signed-rank test, *P* < 0.05, FDR corrected). Furthermore, recurrent DNNs showed an advantage compared with shallow feedforward DNNs for the classification of location with high clutter and for the prediction of location representations in LOC (Supplementary Fig. 3c,d; *N* = 25, 4 × 2 repeated-measures ANOVA). Together, these results suggest that location information of objects on highly cluttered scenes emerges in LOC with local recurrent processes.

### Object category representations

**Fig. 6:**
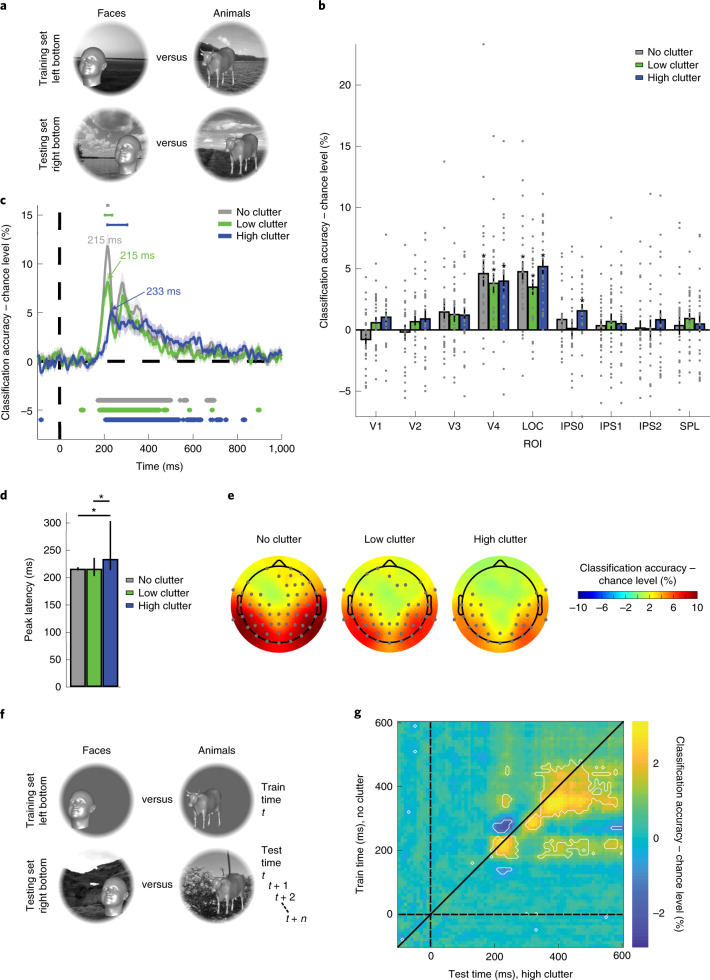
Spatial and temporal dynamics of object category representations. **a**, Classification scheme of category across location. **b**, Location-tolerant category representations in the ventral and dorsal streams. Stars indicate classification above chance level (two-tailed Wilcoxon signed-rank test, *P* < 0.05, FDR corrected). Conventions as in Fig. 3b. **c**, Results of the time-resolved category classification across locations from EEG activation patterns. Conventions and statistics as in Fig. 5a. **d**, Peak latencies of curves in **c**. Statistics and conventions as in Fig. 5b. **e**, Results of searchlight in EEG channel space at peak latencies in no-, low- and high-clutter condition, down-sampled to 10 ms steps. Significant electrodes are marked in grey (*N* = 27, two-tailed Wilcoxon signed-rank test, *P* < 0.05, FDR corrected across electrodes and time points). **f**, Time generalization analysis scheme for classifying object category across location and background condition. **g**, Results of the time generalization analysis (*N* = 27, two-tailed Wilcoxon signed-rank test, *P* < 0.05, FDR corrected). Conventions as in Fig. 5e.

The observation that representations of object location depend on the background on which the object appears immediately raises the question of whether representations of object category are affected by background, too. Previous research suggests opposite answers to this question. One line of research demonstrated that object representations in the ventral stream are modulated by the presence of other objects and the background on which they are viewed^40–43^. Another line of research has provided strong evidence that the ventral stream constructs object representations that are increasingly tolerant to changes in viewing conditions^1,5,8^, suggesting that object category representations should be unaffected by the background of the objects. Here we bring these two lines of research together by explicitly investigating how background impacts object category representations that are tolerant to location. To do this, we analysed EEG and fMRI data as described in previous sections but exchanging the role of experimental factors location and category. In essence, we performed cross-classification analyses of category across location (Fig. 6a) to determine where and when location-tolerant object category representations emerge in the human brain.

#### The locus of object category representations

We investigated object category representations tolerant to changes in location using an ROI-based fMRI analysis. We observed that location-tolerant object category could be classified in the ventral stream in V4 and LOC (Fig. 6b*; N* = 25, two-tailed Wilcoxon signed-rank test, *P* < 0.05, FDR corrected, all *P* values in Supplementary Table 1), but not at earlier stages and not in dorsal ROIs except IPS0 with high clutter (*P* = 0.005). This pattern was not influenced by the level of clutter, suggesting that object category representations that are tolerant to location variations are unaffected by the clutter level of the background on which the object appears.

These observations were statistically ascertained in a 5 × 3 ANOVA along the ventral stream with factors ventral ROIs (V1, V2, V3, V4 and LOC) and background (no, low and high clutter), revealing a significant main effect of ROI (*F*_(2.42,58.03)_=21.97, *P* < 0.001, partial *η*^2^ = 0.48), but not of background (*F*_(2,48)_ = 0.68, *P* = 0.510) and no interaction (*F*_(8,192)_=1.85, *P* = 0.070, see Supplementary Fig. 8 for searchlight results and Supplementary Table 7 for post hoc tests, Tukey corrected). In the 7 × 3 repeated-measures ANOVA along the dorsal stream with factors ROI (V1, V2, V3, IPS0, IPS1, IPS2 and SPL) and background (no, low and high clutter) we found no significant main effect (ROI: *F*_(6,144)_=1.38, *P* = 0.227; background: *F*_(2,48)_ = 0.94, *P* = 0.396) or interaction effect (*F*_(12,288)_ = 0.96, *P* = 0.463).

In sum, our results confirm that the ventral stream constructs object representations that are robust to changes in viewing conditions and show in particular that location-tolerant category representations emerge in the ventral stream unaffected by the clutter level in the object’s background.

#### Object category representations in time

Emergence of object category representations can be delayed, for example when objects are occluded or are hard to categorize^44–46^. This suggests that object category representations might emerge with a delay also when objects appear on cluttered backgrounds, for example because additional grouping and segmentation operations are necessary that depend on recurrence and hence require additional time^47–49^.

We therefore investigated whether background clutter influences the timing with which location-tolerant category representations emerge using time-resolved multivariate EEG analysis (Fig. 6c). We found that object category could be reliably classified for all background conditions from the EEG data (Fig. 6c, *N* = 27, two-tailed Wilcoxon signed-rank test, *P* < 0.05, FDR corrected), but with distinct temporal dynamics (see Supplementary Table 5 for classification onsets and peak values). Classification peaks were 18 ms later in the high-clutter than in the no- and the low-clutter conditions (no clutter: 215 ms (213–219 ms); low clutter: 215 ms (203–236 ms); high clutter: 233 ms (214–303 ms)). The delay (95% difference CI no clutter: 16–173 ms; *P* < 0.001; low clutter: 13–171 ms; *P* = 0.029) was significant (*N* = 27, bootstrap test, 10,000 bootstraps, *P* < 0.05, one-tailed bootstrap test against zero, FDR corrected; Fig. 6d). Location-independent category information at the peaks of the three background conditions was most pronounced at occipital and temporal electrodes as revealed in the EEG searchlight in sensor space (Fig. 6e and Supplementary Fig. 5g–i; *N* = 27, two-tailed Wilcoxon signed-rank test, *P* < 0.05, FDR corrected across electrodes and time points). This is in line with the results from the fMRI searchlight analysis (Supplementary Fig. 8), together suggesting neural sources of the peaks in Fig. 6c in occipital and temporal regions. Univariate EEG activity was strongest in occipital rather than in temporal electrodes (Supplementary Fig. 5,j,k,l). Together, this shows that cortical processing of object category requires more time when objects appear in cluttered scenes compared with artificial blank backgrounds.

Analogous to the delay in location processing (Fig. 5a,b,e), we asked whether this delay indicates a temporal shift in the processing cascade or reflects a change to a later processing stage. To disambiguate, we classified object category across locations in a time generalization analysis across the no- and the high-clutter conditions (Fig. 6f).

We identified three main clusters of high classification accuracy with timing corresponding roughly to the timing of the three peaks observed in the time courses of the no- and the high-clutter conditions (Fig. 6g; see Supplementary Table 8 for timing details). To test whether category information in the no-clutter condition generalized to later time points in the high-clutter condition and thus was shifted below the diagonal, we computed the single-subject distances from the peak in the time generalization matrix to the diagonal. Category information peaks were significantly shifted below the diagonal as tested across subjects (mean Euclidean distance 27.24 ms; *N* = 27, two-sided Wilcoxon signed-rank test, *P* = 0.025, *r* = 0.43, *s.e.m.* 2.50; single-subject peaks shown in Supplementary Fig. 6c), but not as tested for the group-averaged peak (Euclidean distance 28.28 ms; 10,000 bootstraps; one-tailed bootstrap test against zero, *P* = 0.230; 95% CI −7.07 to 35.35). Classification accuracies were significantly higher below than above the diagonal from ~190 ms (no clutter) and ~240 ms (high clutter) until ~360 ms (no clutter) and ~400 ms (high clutter) (Supplementary Fig. 6d). This pattern of results suggests that object category representations of objects on blank and cluttered backgrounds emerge at a similar processing stage. This stage emerges with a delay when objects are presented on cluttered backgrounds, indicating recurrent processing.

## Discussion

Using multivariate analysis of fMRI and EEG data and computational model comparison, we resolved where, how and when object location representations emerge in the human brain. Our results are three fold and depend crucially on whether objects appeared on cluttered backgrounds or on blank backgrounds. First, location representations emerged along the ventral visual pathway and peaked in region LOC when viewed on cluttered backgrounds. Second, this pattern of results was mirrored in DNNs trained on object categorization. Third, location representations emerged later in time when objects were viewed on cluttered backgrounds than when viewed on blank backgrounds. In-depth analysis suggested that this delay indexed recurrent processing in LOC. Together, these results provide converging evidence against the hypothesis that object location is processed in early visual cortex (H1), and in addition the results in space provide evidence for the hypothesis that object location emerges along the ventral stream (H3, Fig. 1a). A corresponding analysis of object category representations revealed equivalently an emergence in the ventral visual stream, and a delay when objects appear on cluttered backgrounds due to a temporal shift in the processing cascade, related to recurrent processing. Thus, the two arguably most fundamental properties of objects, that is, what the object is and where it is, emerge in the ventral visual stream with a similar spatiotemporal processing pattern.

Our fMRI results single out the ventral stream with a peak in LOC (H3), rather than early visual areas (H1) or the dorsal stream (H2), as the processing hierarchy responsible for computing object location in the human brain when objects appear on cluttered backgrounds. This concurs with a primate study^14^ that found category-orthogonal object representations to emerge in IT (the putative homologue of human LOC^50^) rather than V4. Together, these results indicate that object location representations emerge along the ventral stream towards LOC when viewing conditions are realistic and challenging.

We observed that location representations with high clutter increased along the ventral stream for the classification of cross- but not within-hemifield locations. This pattern of results might be due to several factors. For one, statistical power is reduced when assessing results of cross- and within-hemifield location classification separately rather than combined, the test for which our study was originally planned. Second, cross-hemifield location representations might be more distinguishable as there is less integration of location information across than within hemispheres: cross-hemifield integration requires trans-callosal connections, whereas within-hemifield integration does not. Third, factors unrelated to location representations that however affect hemispheres differently, such as possible vascular changes, can contribute to the effect. Importantly, we do not see a difference between within- versus across-hemifield classification in the high-clutter condition in the EEG and DNN results, supporting our main conclusions and suggesting that the discrepancy in the fMRI results might be related to a decreased signal-to-noise ratio.

When objects are viewed on blank backgrounds rather than on cluttered backgrounds, location information can be read out from V1 because there is a direct mapping from stimulus location to the retinotopic location in V1 that is activated. With clutter, there is no such mapping (Fig. 1b) and therefore visual input is processed through the ventral visual stream cascade where LOC but not V1 reliably indicates object location representations. Under this assumption, location information in V1 might be an epiphenomenon caused by artificial stimulation conditions, revealing information that can be measured by the experimenter but is not necessarily used by the brain^51–53^ and relevant for behaviour at this stage of processing. Our results thus further emphasize the importance of increasing image complexity to increase the ecological validity of experimental stimuli^21^. While our study was designed to establish the presence and nature of object location representations in the brain, it cannot establish the behavioural relevance of those representations. Future studies could investigate this, for example, by using speeded detection tasks for objects presented in different locations and relating detection speed and performance to location representations across the brain.

Our results are seemingly at odds with neuropsychological findings showing that patients with ventral lesions performed well on localization tasks^2^. However, later studies showed that in fact just localization behaviour was intact in those patients^54–56^, but not location perception. It is conceivable that these patients recruited sparse location information from spared early visual areas to accomplish the localization tasks (similar to blindsight) and that tasks involving more cluttered displays would have been more challenging for these patients. In line with this, other patients with occipito-temporal lesions had problems with tasks requiring figure–ground segmentation^57^ or perceptual grouping^58^, both of which are essential to dissect an object from its background in a cluttered scene. Thus, neuropsychological studies taking background clutter into account are necessary to resolve this issue.

While we do observe location information in dorsal and ventral regions anterior and medial from LOC, the fMRI searchlight analysis (Supplementary Fig. 2) shows the peak in LOC. Why did location information not peak in other high-level ventral or dorsal areas? It is possible that IPS would represent object location more prominently if we optimized our stimulus selection for it by including tools^51^. However, the univariate response profile of the dorsal and ventral ROIs in our study tentatively suggests comparable activations across ROIs (Fig. 4d and Supplementary Table 4), indicating that univariate activation was not the source of lower information in IPS. Likewise, it is possible that different stimuli (for example, faces) would have yielded stronger effects in other high-level, category-selective ventral regions (for example, fusiform face area or occiptal face area). Another possibility is that LOC has optimal receptive field properties for the eccentricities used in this study^59,60^, which allows it to encode object location on clutter better than other high-level ventral ROIs. These questions need more investigation in future research.

Our empirical findings were reinforced by the observation that representations of object location emerge in DNNs in a similar way as they emerge in the human brain. Importantly, the DNNs used here were trained on object categorization and not localization. Our results thus show that representations of object properties for which the network is not optimized can emerge in such networks^14^. One limitation of our approach is that the models used here were specifically designed to model the ventral visual stream^25–30^, even though they have been shown to predict brain responses in the dorsal stream, too^32,33^. Therefore, the presented modelling results cannot distinguish between H2 and H3. Future studies could compare location representations in DNNs that model the dorsal versus the ventral stream and investigate how the model’s representations relate to brain representations in the two streams.

The time-resolved EEG analyses and the EEG–fMRI fusion analysis^38^ revealed together that location representations of objects with high clutter were delayed due to a temporal shift within the same processing stage in LOC. Since temporal delays at the same processing stage cannot be explained purely by a feedforward neural architecture, this indicates the involvement of recurrence. Physiologically, this might be implemented via lateral connections within LOC, resulting in slower information accumulation^61,62^. Furthermore, we found not only location but also object category representations to be delayed when objects were superimposed on natural scenes. Together with previous reports that object category processing can be delayed when objects are degraded, occluded or are hard to categorize^44–46,48^, our results add to the emergent view that recurrent computations are critically involved in the processing of fundamental object properties such as what objects are^62^ and where they are in real world vision. Future studies could provide more direct evidence for recurrence by manipulating it experimentally, for example, by adding a masking condition to the study design used here.

We find that both object category and object location representations emerged gradually along the ventral visual stream. This might seem counter-intuitive, given that transformations that lead to the emergence of category representations in LOC have been linked to building increasing tolerance to viewing conditions, in particular to changes in object location^5–7^. However, this apparent contradiction is qualified by the observation that the observed tolerance to changes in viewing conditions is graded rather than absolute^63^, mirrored by the presence of cells in high-level ventral visual cortex with large overlapping receptive fields^10,17^. Such tuning properties provide the spatial resolution needed for localization^64^, while also providing robustness to location translation^65^, needed for object categorization.

In this study, we deliberately avoided congruence between objects and backgrounds, which is known to lead to interaction effects with category processing^40^. However, this deviation from normality in our stimulus set might have triggered mismatch responses that lead to additional recurrent processing for disambiguation or attentional responses triggered by atypical object appearance (for example, size and texture). Further, because objects and backgrounds did not form a coherent scene, objects and backgrounds might have been represented more independently. Another design limitation is that we constrain the number of locations to four to fully cross all stimulus conditions while maintaining a feasible session duration. Future research will have to establish whether congruent versus incongruent scene–object pairings yield different location representations on cluttered backgrounds and whether our results generalize to more locations.

What an object is and where an object is are arguably the two most fundamental properties that we need to know to be able to interact with objects in our environment. Our results reveal the basis of this knowledge by revealing representations of location and category in the human brain when viewing conditions are challenging, as encountered outside of the laboratory. Both object location and category representations emerge along the ventral visual stream towards LOC and depend on recurrent processing. Together, our results provide a spatiotemporally resolved account of object vision in the human brain when viewing conditions are cluttered.

## Methods

### Participants in EEG and fMRI experiments

The experiment was approved by the ethics committee of the Department of Education and Psychology of the Freie Universität Berlin (ethics reference number 104/2015) and was conducted in accordance with the Declaration of Helsinki. Twenty-nine participants participated in the EEG experiment, of whom two were excluded because of equipment failure (*N* = 27, mean age 26.8 years, *s.d.* 4.3 years, 22 female). Twenty-five participants (mean age 28.8 years , s.d. 4.0 years, 17 female) completed the fMRI experiment. The participant pools of the experiments did not overlap except for two participants. Sample size was chosen to exceed comparable magnetoencephalography, EEG and fMRI classification studies to enhance power^8,9,43,66–68^. All participants had normal or corrected-to-normal vision and no history of neurological disorders. All participants provided informed consent prior to the studies and received a monetary reward or course credit for their participation.

### Experimental design

To enable us to investigate the representation of object location, category and background independently, we used a fully crossed design with factors of category (four values: animals, cars, faces and chairs; Fig. 2a, left, with three exemplars per category), location (four values: left up, left bottom, right up and right bottom; Fig. 2a left centre) and background clutter (three values: no, low and high clutter; Fig. 2a, right centre). This amounted to 144 individual condition combinations (12 object exemplars × 4 locations × 3 background clutter levels). We analysed the data at the level of category, effectively resulting in 48 experimental conditions (4 categories × 4 locations × 3 background clutter levels).

### Stimulus set generation

The stimulus material was created by superimposing three-dimensional (3D) rendered objects (Fig. 2a, left) with Gouraud shading in one of four image locations (Fig. 2a, left centre) onto images of real-world backgrounds (Fig. 2a, right centre).

In detail, in each category, one of the objects was rotated by 45°, one by 22.5° and the third by −45° with respect to the frontal view to introduce equal variance in the viewing angle for each category. Locations were in the four quadrants of the screen (Fig. 2a, left centre). Expressing locations in degrees of visual angle, the object’s centre was 3° visual angle away from the vertical and horizontal central midlines (that is, 4.2° from image centre; Fig. 2a, right). The size of the objects was adjusted so that all of them fitted into one quadrant of the aperture, while maintaining a similar size (mean (*s.d.*) size: vertical, 2.4° (0.4°); horizontal, 2.2° (0.6°)).

We used backgrounds with three different clutter levels: no, low and high (Fig. 2a, right centre; note that example backgrounds shown here are for illustrative purposes and were not used in the experiment. The original stimulus material is available for download together with the data). We defined clutter as the organization and quantity of objects that fill up a visual scene^69^. In the no-clutter condition, the background was uniform grey. In the low- and the high-clutter condition, we selected a set of 60 natural scene images each from the Places365 database (http://places2.csail.mit.edu/download.html) that had low or high clutter, respectively, and did not contain objects of the categories defined in our experimental design (that is, no animals, cars, faces or chairs). We converted the images to greyscale and superimposed a circular aperture of 15° visual angle. The visual angle was the same in the EEG and fMRI experiments.

We confirmed that our selection of low- and high-clutter images was appropriate by an independent behavioural rating experiment (*N* = 10) in which participants rated clutter level on a scale from 1 to 6 (mean (*s.d.*) clutter image rating: low clutter, 2.52 (0.85); high clutter, 5.04 (0.87); the difference was significant: *N* = 10, paired-sample *t* test, *P* < 0.0001, *t* = 14.96).

From the set of 60 low- and high-clutter images, we selected 48, one for each experimental condition of our experimental design. We then randomly paired objects to background images to avoid systematic congruencies between backgrounds and objects. This was done for each of the 20 runs of the EEG experiment and for the 10 runs of the fMRI experiment. This resulted in 144 individual images per run, one for each condition (that is, 12 object exemplars × 4 locations × 3 background clutter levels). The remaining set of 12 low- and high-clutter images was used separately to create catch trials in the EEG experiment (see details below).

### Experimental procedures

#### fMRI main experiment

Each participant completed one fMRI recording session consisting of ten runs (run duration 552 s), resulting in 92 min of fMRI recording of the main experiment. During each run, each of the 144 images of the stimulus set was shown once (denoted here as ‘regular’ trials) in random order. Image duration was 0.5 s, with a 2.5 s inter-stimulus interval (ISI). Images were presented at the centre of a black screen, overlaid with a red fixation cross in the centre. Participants were asked to fixate their eyes on the central cross at all times. Regular trials were interspersed every third to fifth trial (equally probable, in total 36 per run) with catch trials. Catch trials repeated the image shown on the previous trial (Fig. 2b, bottom). Participants were instructed to respond with a button press to these repetitions (that is, a one-back task). Catch trials were excluded from further analysis. Since this was a repeated-measures design, data collection and analysis were not performed blind to the conditions of the experiment.

#### fMRI localizer experiment

To define ROIs in early visual, dorsal and ventral visual stream areas, we performed a separate localizer experiment prior to the main fMRI experiment with images in three experimental conditions: faces, objects and scrambled objects. Each image shown in the localizer experiment consisted of four identical versions of an object presented at the four locations as defined in the main experiment (for example, one particular face shown in all four quadrants) to approximate the stimulation conditions of the main experiment.

The localizer experiment consisted of a single run lasting 384 s, comprising six blocks of presentation of faces, objects, scrambled objects and a blank background as baseline. Each stimulation block was 16 s long with presentations of 20 different objects (500 ms on, 300 ms off), including two one-back repetitions that participants were instructed to respond to with a button press. Stimulation block order was first order counterbalanced, with triplets of stimulation blocks being presented in random order and being interspersed regularly with blank background blocks.

#### EEG main experiment

The EEG experiment was a modified version of the fMRI main experiment with adjusted timing parameters and a different task (Fig. 2b, top). The EEG recording session consisted of 20 runs of 205 s each (that is, in total 68 min). Twenty-three participants completed all 20 runs, while four participants completed fewer runs due to technical problems (12 runs, 17 runs and 2 × 13 runs). Image duration was 0.5 s, with a 0.5 or 0.6 s ISI (equally probable) on regular trials. Participants were asked to fixate their eyes on the central cross at all times. Catch trials consisted of the presentation of the target object (a glass) at any of the four locations and on any type of background. Participants were instructed to respond with a button press to the glass (that is, a detection task), and to blink their eyes to minimize eye blink contamination on regular trials. To avoid contamination of movement and eye blink artefacts on subsequent trials, the ISI was 1 s on catch trials. Catch trials were excluded from further analysis. Since this was a repeated-measures design, data collection and analysis were not performed blind to the conditions of the experiment.

### Pre-processing and univariate fMRI analysis

#### fMRI acquisition and pre-processing

We acquired MRI data on a 3-T Siemens Tim Trio scanner with a 12-channel head coil. We obtained a structural image using a T1-weighted sequence (magnetization-prepared rapid gradient-echo, 1 mm^3^ voxel size). For the main experiment and the localizer run, we obtained functional images covering the entire brain using a T2*-weighted gradient-echo planar sequence (repetition time 2 ms, echo time 30 ms, 70° flip angle, 3 mm^3^ voxel size, 37 slices, 20% gap, 192 mm field of view, 64 × 64 matrix size, interleaved acquisition).

We pre-processed fMRI data using SPM8 (https://www.l.ion.ucl.ac.uk/spm/). This involved realignment, coregistration and normalization to the structural Montreal Neurological Institute template brain. fMRI data from the localizer was smoothed with an 8 mm full-width at half-maximum Gaussian kernel, but the main experiment data was left unsmoothed.

#### Univariate fMRI analysis

For the main experiment, we modelled the fMRI responses to the 48 experimental conditions for each run using a general linear model (GLM). The onsets and durations of each image presentation entered the GLM as regressors and were convolved with a haemodynamic response function. Movement parameters entered the GLM as nuisance regressors. For each of the 48 conditions, we converted GLM parameter estimates into *t* values by contrasting each parameter estimate against the implicit baseline. This resulted in 48 condition-specific *t* value maps per run and participant.

For the localizer experiment, we modelled the fMRI response to the three experimental conditions, entering block onsets and durations as regressors of interest and movement parameters as nuisance regressors before convolving with the haemodynamic response function. From the resulting three parameter estimates, we generated two contrasts. The first contrast served to localize activations in early, mid-level ventral and dorsal visual regions (V1, V2, V3, V4, IPS0, IPS1, IPS2 and SPL) and was defined as objects + scrambled objects > baseline. The second contrast served to localize activations in object-selective area LOC and was defined as objects > scrambled objects. In sum, this resulted in two *t* value maps for the localizer run per participant.

#### Definition of ROIs

To identify regions along the ventral and dorsal visual streams, we defined ROIs in a two-step procedure. We first defined ROIs using anatomical masks from a probabilistic atlas^70^ for both hemispheres combined (three early visual ROIs for regions shared between the ventral and dorsal stream (V1, V2 and V3), two ROIs in mid- and high-level ventral visual cortex (V4 and LOC) and four ROIs in dorsal visual cortex (IPS0, IPS1, IPS2 and SPL)). To avoid overlap between the ROI masks we removed all overlapping voxels. In a second step we selected the 325 most activated voxels of the participant-specific localizer results within the masks, using the objects > scrambled contrast for LOC and the objects & scrambled objects > baseline contrast for the remaining ROIs. This yielded participant-specific ROI definitions.

### EEG acquisition and pre-processing

We recorded EEG data using an EASYCAP 64-channel system and a Brainvision actiCHamp amplifier at a sampling rate of 1,000 Hz. The electrodes were placed according to the standard 10–10 system. The data were filtered online between 0.03 and 100 Hz and re-referenced online to FCz.

Offline pre-processing was conducted using the EEGLAB toolbox (version 14)^71^ and incorporated a low-pass filter with a cut-off at 50 Hz and epoching trials between −100 ms and 999 ms with respect to stimulus onset. Epochs were baseline corrected by subtracting the mean of the 100 ms prestimulus time window from the entire epoch. To clean the data from artefacts such as eye blinks, eye movements and muscular contractions, we used independent component analysis as implemented in the EEGLAB toolbox. SASICA^72^ was used to guide the visual inspection of components for removal. Components related to horizontal eye movements were identified using two lateral frontal electrodes (F7 and F8). In the last six participants, additional external electrodes were available that allowed for the direct recording of the horizontal electro-oculogram to identify and remove components related to horizontal eye movements. For blink artefact detection based on the vertical electro-oculogram, we used two frontal electrodes (Fp1 and Fp2). On average, 11 (*s.d.* 4) components were removed per participant. As a final step, we applied multivariate noise normalization to improve the signal-to-noise ratio and reliability of the data (following the recommendation of Guggenmos et al.^73^).

### Object location classification from brain measurements

To determine the amount of location information independent of category present in multivariate brain measurements, we applied a common multivariate cross-classification scheme^8,66–68^. In essence, separately for each background condition, we classified location while assigning data from different object categories to the training and testing sets (Supplementary Fig. 1a). All classification analyses relied on binary c-support vector classification with a linear kernel as implemented in the libsvm toolbox^74^ (https://www.csie.ntu.edu.tw/cjlin/libsvm). Furthermore, all analyses were conducted in a participant-specific manner.

#### Spatially resolved multivariate fMRI analysis

We conducted an ROI-based and a spatially unbiased volumetric searchlight procedure^24,75^. For the ROI-based analysis, for each ROI separately, we extracted and arranged *t* values into pattern vectors for each of the 48 conditions and 10 runs. To increase the signal-to-noise ratio, we randomly binned run-wise pattern vectors into five bins of two runs, which were averaged, resulting in five pseudo-run pattern vectors. We then performed five-fold leave-one-pseudo-run-out cross-validation, training on four and testing on one pseudo-trial per classification iteration. In detail, we assigned four pseudo-trials per location condition of the same category to the training set (Supplementary Fig. 1a). We then tested the SVM on one pseudo-trial for each of the same two location conditions, but now from a different category, yielding per cent classification accuracy (50% chance level) as output. Equivalent SVM training and testing was repeated for all combinations of location and category pairs. With four locations that were all classified pairwise once, this resulted in six pairwise location classifications. In addition, each pairwise location classification was iterated across all possible training and testing combinations of the four categories. This yielded an additional 12 iterations per location classification across training and testing pairs of categories. Therefore, in total 72 (6 × 12) classification accuracies were averaged during each of the five-fold cross-validation iterations, resulting in 360 averaged accuracies in total. The result reflects how much category-tolerant location information was present for each ROI, participant and background condition separately.

The searchlight procedure was conceptually equivalent to the ROI-based analysis with the difference of the selection of voxel patterns entering the analysis. For each voxel v_*i*_ in the 3D *t* value maps, we defined a sphere with a radius of four voxels centred around voxel v_*i*_. For each condition and run, we extracted and arranged the *t* values for each voxel of the sphere into pattern vectors. Classification of location across category proceeded as described above. This resulted in one average classification accuracy for voxel v_*i*_. Iterated across all voxels, this yielded a 3D volume of classification accuracies across the brain for each participant and background condition separately.

#### Time-resolved classification of location from EEG data

To determine the timing with which category-independent location information emerges in the brain, we conducted time-resolved EEG classification^68,76^. This procedure was conceptually equivalent to the fMRI location classification in that it classified location while assigning data from different categories to the training and testing sets and was conducted separately for each background condition and participant (Supplementary Fig. 1a).

For each time point of the epoched EEG data, we extracted 63 EEG channel activations and arranged them into pattern vectors for each of the 48 conditions and 60 raw trials. To increase the signal-to-noise ratio, we randomly assigned raw trials into four bins of 15 trials each and averaged them into four pseudo-trials. The classification was conducted on those four pseudo-trials. We trained the SVM on three pseudo-trials and tested it on the remaining pseudo-trial, yielding per cent classification accuracy (50% chance level, binary classification) as output. This procedure was repeated 100 times with random assignment of trials to pseudo-trials, and across all combinations of location and all category pairs. As for the fMRI classification, in total 72 (6 location pairs × 12 category train–test pairs) classification accuracies were averaged. With 100 iterations to randomly assign trials to training and testing bins, this yielded a total of 7,200 classification accuracies, which were averaged per background condition and participant. The result reflects how much category-tolerant location information was present at each time point, participant and background condition separately.

#### Time-resolved EEG searchlight in sensor space

We conducted an EEG searchlight analysis resolved in time and sensor space (that is, across EEG channels) to gain insights into which EEG channels contained the highest amount of location information and therefore contributed most to the results of the time-resolved analysis described above. For the EEG searchlight, we conducted the time-resolved EEG classification as described above with the following difference: For each EEG channel c, we conducted the classification procedure on the five closest channels surrounding c. The classification accuracy was stored at the position of c. After iterating across all channels and down-sampling the time points to a 10 ms resolution, this yielded a classification accuracy map across all channels and down-sampled time points, for each participant and background condition separately.

#### Time generalization analysis of location from EEG data

To determine when object location representations are similar across background conditions and time, we used temporal generalization analysis^34,38,68,76^.

The procedure was equivalent to the multivariate time-resolved EEG location classification analysis but with two crucial differences. First, data from the no-clutter condition were assigned to the training set while data from the high-clutter condition were assigned to the testing set (Supplementary Fig. 1b). The second difference was that the SVM was not only tested on data from the same time point as that from which the testing data were derived, but additionally on data from each time point from the −100 to 600 ms peri-stimulus time window (in 10 ms steps). Like previously, training was conducted on three and testing on one pseudo-trial, resulting in 7,200 classification accuracies (6 location pairs × 12 category train–test pairs × 100 randomization iterations), which were averaged per time point and participant. This resulted in a two-dimensional matrix of classification accuracies indicating the combination of time points in the no- and high-clutter conditions at which object location representations were similar in the no- and the high-clutter conditions.

#### Off-diagonal peak shift in time generalization matrix

To quantify whether classification accuracies were significantly higher below than on or above the diagonal, we computed the distance from the post-stimulus classification peak to the diagonal for single subjects. For this, we first determined the peak coordinates (*p*_*x*_, *p*_*y*_) along the *x* and *y* axes. We then computed the coordinates of the point on the diagonal that was closest to the peak using

$$b_x = \frac{{(p_x + p_y)}}{2}$$

since on the diagonal, *b*_*x*_ = *b*_*y*_. This allowed us to compute the shortest perpendicular Euclidean distance between the peak and the diagonal as

$$d_{{\mathrm{Euclidean}}} = \sqrt {(p_x - b_x)^2 + (p_y - b_x)^2}.$$

To be able to later test group distances against zero, we set

$$d_{{\mathrm{Euclidean}}} = d_{\mathrm{Euclidean}} \times - 1$$

for all cases where *p*_*x*_ < *p*_*y*_, which is the case for all peaks above the diagonal.

#### Diagonal difference in temporal generalization matrix

To obtain a temporally resolved estimate of the time points at which the classification accuracy was higher below than above the diagonal, we subtracted the classification accuracies above the diagonal from the accuracies below the diagonal. Specifically, we subtracted each time point from the time point with the equivalent coordinates mirrored along the diagonal. For example, the time point with coordinates 300 ms in the no-clutter (*y* axis) and 100 ms in the high-clutter (*x* axis) condition (above diagonal) was subtracted from the time point with coordinates 100 ms in the no-clutter (*y* axis) and 300 ms in the high-clutter (*x* axis) condition (below diagonal).

### EEG–fMRI fusion

To determine the spatiotemporal correspondence between object location representations revealed at particular time points in the EEG signals and localized in particular cortical regions using fMRI, we used representational similarity analysis-based EEG–fMRI fusion^37–39^. We focused the analysis on representations emerging at peak latencies in the EEG and on ventral stream ROIs. The rationale for this approach is that time points and ROIs are linked if they represent object locations similarly, that is, if their representational geometries (dissimilarity relations between representations) are comparable.

As a measure of (dis-)similarity relations between location representations, we used the classification results from the multivariate analyses conducted. This choice assumes that representations for two locations will be classified more easily if they are more dissimilar. In detail, we considered the pairwise classification accuracies between all pairs of locations (six) and all training and testing pairs across categories (six) in both training and testing directions (two), resulting in a 72 × 1 RDV. For EEG, we extracted the RDVs for the time points within the confidence intervals around the EEG peak latency, averaged them across time points and, following the method employed previously^32,77,78^, averaged them across participants, resulting in one EEG RDV per background condition. For fMRI ROIs, we extracted the RDVs for each participant and background condition separately.

We compared fMRI and EEG RDVs for representational similarity by correlating (using Spearman’s *R*) the averaged EEG RDV with the subject-specific fMRI ROI RDVs, resulting in one correlation per subject, background condition and ROI.

### Multivariate classification of category

We conducted a set of spatially resolved (fMRI: ROI and searchlight), time-resolved and temporally generalized analyses (EEG) of object category. The analyses were equivalent to the procedures described above with the crucial difference that the role of the experimental factors location and category was reversed (Fig. 6a,f).

### Object location classification in DNNs

We investigated whether DNNs trained on object categorization display a similar pattern of gradually emerging location representations along their processing hierarchy as we observed in the human brain.

We selected the DNN CORnet-S for investigation, on the basis of its top performance in predictivity of neural responses in the ventral stream as quantified on the Brain-Score platform^27^. CORnet-S is a shallow recurrent DNN consisting of four computational blocks referred to as areas, analogous to ventral visual areas V1, V2, V4 and IT. Each block consists of four convolutional layers with self-recurrence and a skip connection followed by group normalization and a rectified linear unit. The response of the final IT block is averaged over the entire receptive field and mapped to categories using a fully connected linear decoder.

To investigate the representation of object location in CORnet-S, we performed multivariate pattern analysis analogous to the analysis performed on brain data, classifying object location across category separately for each background condition. For this, we extracted unit activations of the last layer in each block of the DNN after running a forward pass of the stimulus material from the 20 runs of the EEG experiment.

For the top layer of each block, we arranged the unit activations into pattern vectors for each of the 48 conditions and 60 trials. We then proceeded with the analysis as done with the EEG data (Supplementary Fig. 1a). We randomly assigned raw trials into four bins of 15 trials each and averaged them into four pseudo-trials. We trained the SVM on three pseudo-trials and tested it on the remaining pseudo-trial. This procedure was repeated 100 times with random assignment of trials to pseudo-trials, and across all combinations of location and all category pairs before results were averaged. This resulted in one averaged classification accuracy value per top layer of each CORnet-S block and per background condition. The result reflects how much category-tolerant location information was present in CORnet-S.

### Statistical testing

#### Wilcoxon signed-rank test

We performed non-parametric two-tailed Wilcoxon signed-rank tests to test for above-chance classification accuracy at time points in the EEG time courses, in the EEG time generalization matrix, for Euclidean distances from peak to diagonal in the time generalization matrices, for above-chance classification in the ROI and fusion results and for significant voxels in the fMRI searchlight results. In each case, the null hypothesis was that the observed parameter (classification accuracy, correlation or Euclidean distance) came from a distribution with a median of chance-level performance (that is, 50% for pairwise classification and zero correlation or Euclidean distance). The resulting *P* values were corrected for multiple comparisons using false discovery rate (FDR) at 5% level if more than one test was conducted.

#### Bootstrap tests

We used bootstrapping to compute confidence intervals and to determine the significance of peak-to-peak differences in EEG latencies, peak-to-peak distances of fMRI searchlight classification peaks and for the distance from the group-averaged classification peak in the temporal generalization matrix to the diagonal in Figs. 5e and 6g. In each case, we sampled the participant pool 10,000 times with replacement and for each sample calculated the statistic of interest.

For the fMRI searchlight peak distances, we first shuffled condition labels of two background conditions to then generate a distribution of peak distances under the null hypothesis.

To determine whether peak-to-peak Euclidean distances in searchlight classification maps were significantly longer than expected independent of background, we set *P* < 0.05. If the computed *P* value was smaller than this threshold with Bonferroni correction, we rejected the null hypothesis of no peak-to-peak distance.

For the EEG peak-to-peak latency differences, we bootstrapped the latency difference between two background conditions, yielding an empirical distribution that could be compared with zero.

To determine whether peak-to-peak latencies in the EEG time courses were significantly different from zero, we computed the proportion of values that were equal to or smaller than zero and corrected them for multiple comparisons using FDR at *P* = 0.05. To compute 95% confidence intervals for single peak latencies in the EEG time courses, we bootstrapped the peaks for each background condition and determined the 95% percentiles of this distribution.

#### ANOVAs

We ran sets of ANOVAs to test for main effects and the interaction between ROIs along the ventral and dorsal stream and background condition, which we detail below. For all reported ANOVAs, we tested whether the assumption of sphericity had been met using Mauchly’s test. Below, we report the effects for which the assumption of sphericity had been violated and for which the Greenhouse–Geisser estimates of sphericity were used to correct the degrees of freedom. For all remaining effects, the assumption of sphericity had been met.

To test for main effects and the interaction between ROIs along the ventral stream and background condition, we ran two 5 × 3 repeated-measures ANOVAs with within-subject factors of ROI (V1, V2, V3, V4 and LOC) and background (no, low and high clutter). The first ANOVA tested the results of location classification across categories. Mauchly’s test indicated that the assumption of sphericity had been violated for the main effect of background (*P* = 0.003). Therefore, the degrees of freedom were corrected using the Greenhouse–Geisser estimates of sphericity (*ε* = 0.72). The second ANOVA tested the results of category classification across locations. Mauchly’s test indicated that the assumption of sphericity had been violated for the main effect of ROI (*P* < 0.001). The degrees of freedom were corrected using the Greenhouse–Geisser estimates of sphericity (*ε* = 0.61).

To test for main effects and the interaction between ROIs along the dorsal stream and background condition, we ran two 7 × 3 repeated-measures ANOVAs with within-subject factors of ROI (V1, V2, V3, IPS0, IPS1, IPS2 and SPL) and background (no, low and high clutter). The first ANOVA tested the results of location classification across categories. Mauchly’s test indicated that the assumption of sphericity had been violated for the main effect of ROI (*P* < 0.001) and for the interaction (*P* = 0.028). Therefore, the degrees of freedom were corrected using the Greenhouse–Geisser estimates of sphericity (*ε* = 0.53 for the main effect of ROI, *ε* = 0.52 for the interaction). The second ANOVA tested the results of category classification across locations. Mauchly’s test indicated that the assumption of sphericity had been violated for the interaction (*P* < 0.001). The degrees of freedom were corrected using the Greenhouse–Geisser estimates of sphericity (*ε* = 0.59).

To test for main effects and the interaction in the results of the EEG–fMRI fusion, we ran a 5 × 2 repeated-measures ANOVA with factors of ROI (V1, V2, V3, V4 and LOC) and clutter (no, high). The assumption of sphericity had been met for all main and interaction effects.

All post hoc tests were conducted using pairwise *t* tests, and *P* values were corrected for multiple comparisons using Tukey correction.

#### Effect sizes

For the main and interaction effects of the ANOVAs, we computed the partial *η*^2^ using

$${{{\mathrm{Partial}}}}\,\eta^{2} = \frac{{{\mathrm{Sum}}\,{\mathrm{of}}\,{\mathrm{squares}}\,({\mathrm{SS}})_{{\mathrm{Effect}}}}}{{{\mathrm{SS}}_{{\mathrm{Effect}}} + {\mathrm{SS}}_{{\mathrm{Residual}}}}}$$

and the effect size estimate *r* (ref. ^79^) for the off-diagonal peak shifts across subjects, as tested with the Wilcoxon signed-rank test, using

$$r = \frac{Z}{{\sqrt N }}.$$

### Reporting Summary

Further information on research design is available in the Nature Research Reporting Summary linked to this article.

## Supplementary information

Supplementary information Supplementary Figs. 1–8 and Tables 1–8.

Reporting summary

## Data Availability

The experimental stimuli, fMRI data, EEG data and the neural network activations are publicly available via https://osf.io/7zswn/?view_only=21a714db58584ffeb2837fc0548bf659.

## Code availability

Analysis code is publicly available via https://github.com/graumannm/ObjectLocationRepresentations.
