# Supplementary material for: The spatiotemporal neural dynamics of object location representations in the human brain
Source: Nat Hum Behav. 2022 Feb 24;6(6):796–811. doi: 10.1038/s41562-022-01302-0 (PMC9225954; doi:10.1038/s41562-022-01302-0)
Supplement: Supplementary file 1 — Supplementary Figs. 1–8 and Tables 1–8. [file 41562_2022_1302_MOESM1_ESM.pdf]

nature portfolio

https://doi.org/10.1038/s41562-022-01302-0

Supplementary information

The spatiotemporal neural dynamics of object location representations in the human brain

In the format provided by the authors and unedited

Supplementary Material to

The spatiotemporal neural dynamics of object location representations in the human brain

Monika Graumann[1,2,*], Caterina Ciuffi[1], Kshitij Dwivedi[1,3], Gemma Roig[3], Radoslaw Martin Cichy[1,2,4,*]

1 Department of Education and Psychology, Freie Universität Berlin, 14195 Berlin, Germany

2 Berlin School of Mind and Brain, Humboldt- Universität zu Berlin, 10117 Berlin, Germany

3 Department of Computer Science, Goethe Universität, 60325 Frankfurt am Main, Germany

4 Bernstein Center for Computational Neuroscience Berlin, 10115 Berlin Germany

*Correspondence to:
rmcichy@zedat.fu-berlin.de
monika.graumann@fu-berlin.de

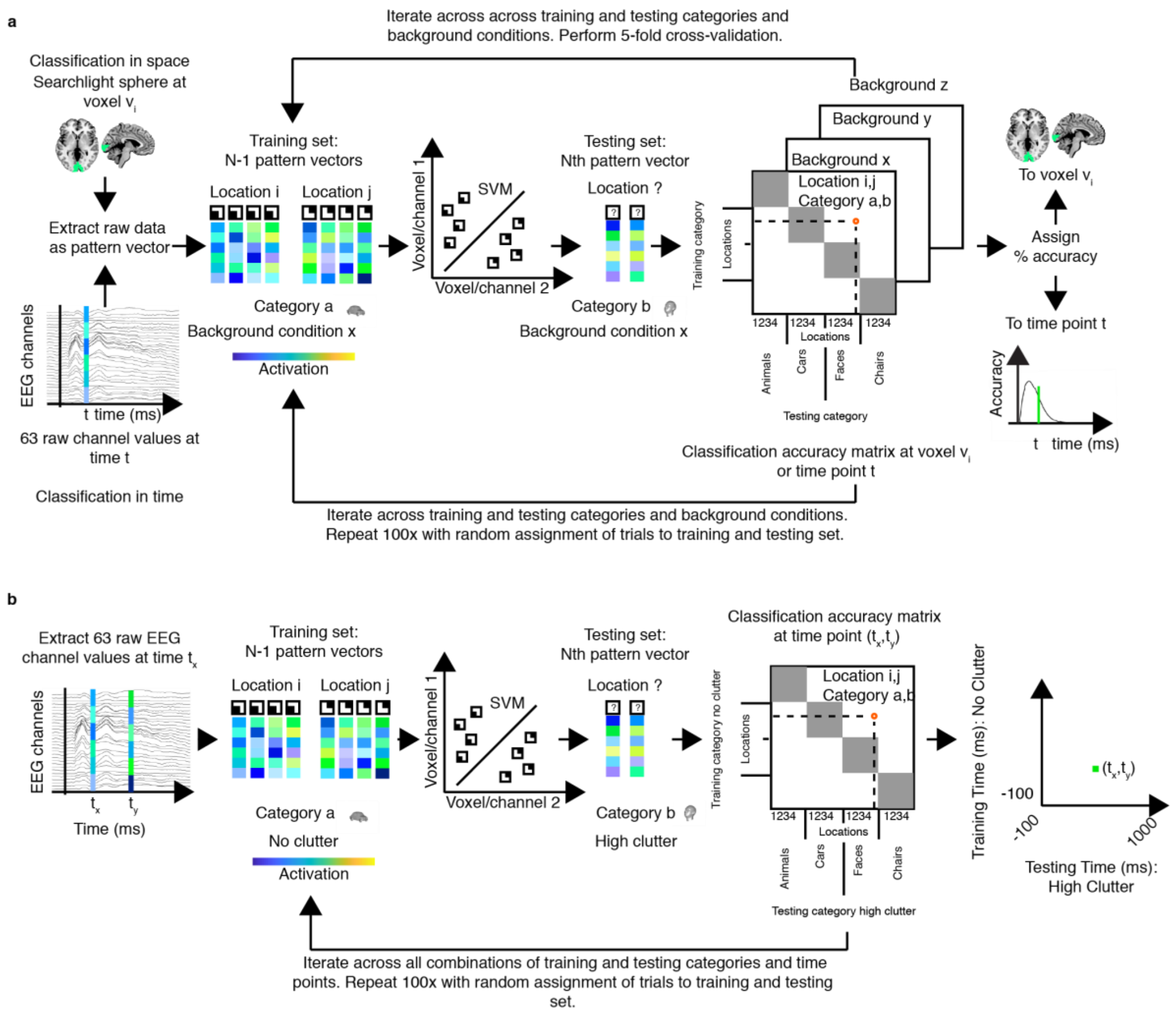

Supplementary Figure 1. Scheme for location classification across categories. a, We used a common multivariate scheme to determine the amount of location information emerging in a spatially-resolved analysis of fMRI data and a temporally-resolved analysis of EEG data. For fMRI data analysis, we extracted pattern vectors of t-values for each experimental condition within a searchlight sphere at voxel v_i or in an ROI. For EEG, we extracted pattern vectors from the 63 preprocessed channel activations for each experimental condition at every time point of the epoch. Then for fMRI and EEG, we divided the pattern vectors into a training and a testing set. We trained a support vector machine (SVM) to pairwise classify between pattern vectors related to the presentation of one object category, presented in two locations (here: cars, left bottom vs. right top). We then tested the SVM on the left-out pattern vectors related to the presentation of a new object category in the same two locations as the training set (here: faces, left bottom vs. right top). This procedure was repeated for all combinations of locations and for all combinations of training and testing categories and this was performed separately in each background condition. Classification accuracies were stored in a 16 × 16 × 3 matrix (4 locations × 4 categories × 3 backgrounds). The upper and lower diagonals contained the location classification accuracies for training and testing in both category directions respectively (i.e., the matrix was not symmetric across the diagonal). The diagonal was undefined because here, training and testing categories were the same. All previous steps were repeated (5-fold cross-validation in fMRI, 100 permutations in EEG) with random assignment of trials to training and testing set, and the resulting classification accuracies were averaged across repetitions, resulting in one classification accuracy value per ROI, voxel or EEG time point. **b,** Time-generalization analysis of location across categories and backgrounds. The classification procedure of location across categories was analogous to (**a**) with two distinctions. First, data from the no clutter condition was always assigned to the training set and data from the high clutter condition was assigned to the testing set. Second, training and testing was repeated across all combinations of time points, which resulted in a two-dimensional matrix.

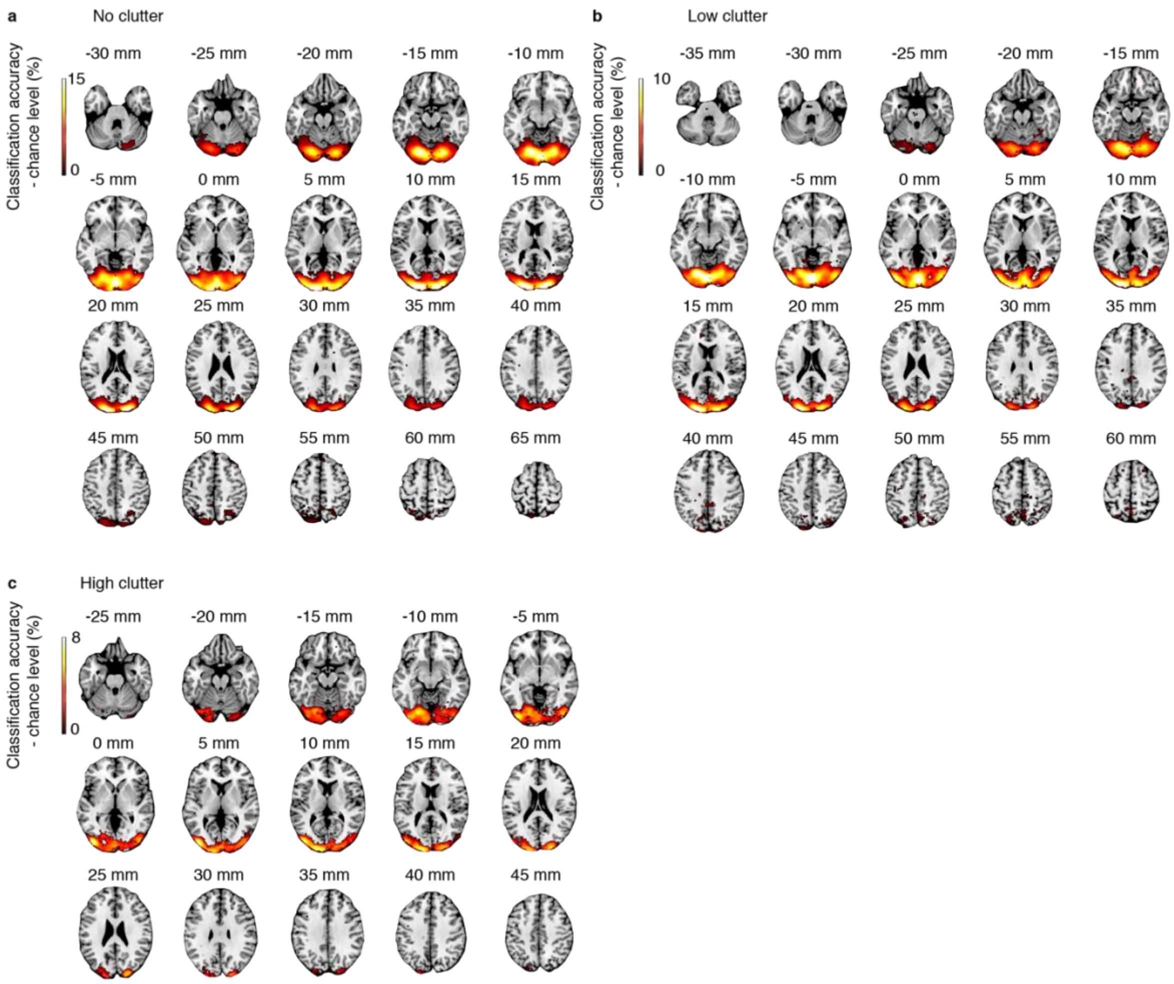

Supplementary Figure 2. Axial slices of the searchlight location classification across category in the a, no clutter b, low clutter c, and high clutter conditions. Colored areas indicate significant location classification across category within that background condition (*N*=25, two-tailed Wilcoxon-signed-rank test, $P<0.05$, FDR-corrected). Millimeters (mm) indicate axial slice position along z-axis in MNI space.

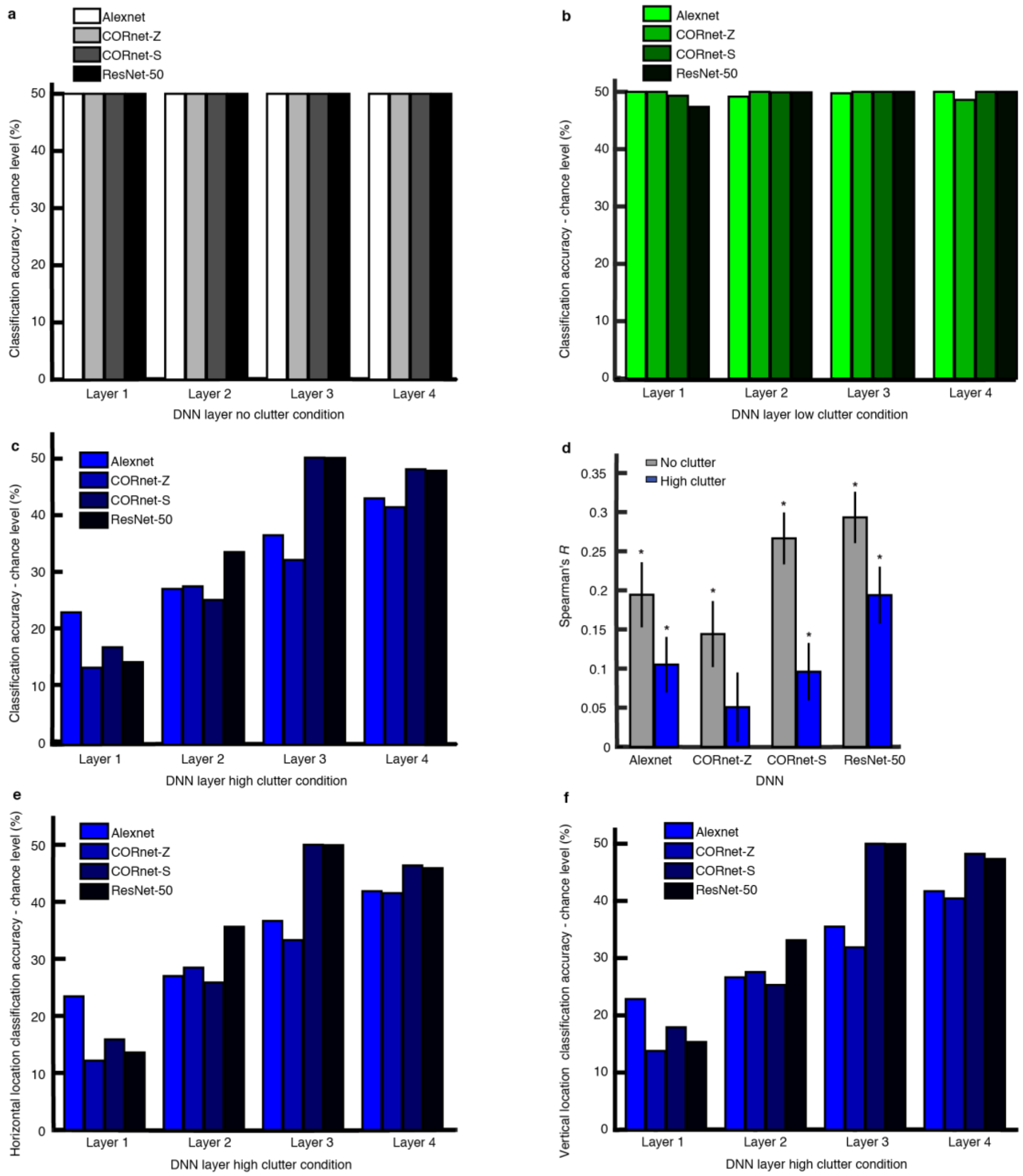

Supplementary Figure 3. Category-independent location representations in four DNN models. We compared two shallow feedforward DNNs (Alexnet, CORnet-Z) to a recurrent DNN (CORnet-S) and a deep feedforward DNN (ResNet-50), which has been shown to be equivalent to an "unfolded" recurrent DNN[1]. **a**, Classification of location across categories in the no clutter condition. For comparability to the other DNNs, we plot convolutional layers 2-5 in Alexnet. Location information was at ceiling in all DNNs and layers. **b**, Same as **a** but in the low clutter condition. Classification accuracy was at or close to ceiling in most layers and DNNs. **c**, In the high clutter condition, all DNNs mirror the increase in location information observed in the ventral stream. However, location information is higher in the recurrent (CORnet-S) and in the deep feedforward (ResNet-50) DNN compared to the shallow feedforward DNNs in higher layers. This demonstrates an advantage of recurrence for location classification when high clutter was present in the images. **d**, Spearman's correlation between category-independent location RDM's in the top layers of each DNN (cross-validated Pearson distance) and the equivalent RDM of LOC (classification accuracies) in the no and high clutter conditions. All correlations reached statistical significance except for the correlation between LOC and CORnet-Z in the high clutter condition (N=25, two-tailed Wilcoxon-signed-rank test, P<0.05, FDR-corrected). A 4 × 2 repeated-measures ANOVA with factors DNN (Alexnet, CORnet-Z, CORnet-S, ResNet-50) and clutter (no, high) revealed a main effect of DNN ($F_{(1.88, 45.10)}=15.96$, $P<0.001$, partial $\eta^2=0.07$) and of clutter ($F_{(1, 24)}=8.55$, $P=0.007$, partial $\eta^2=0.08$) but no significant interaction ($F_{(3,72)}=1.28$, $P=0.29$). Post-hoc tests revealed higher correlations in ResNet-50 than in the other three

DNNs. Correlations were also higher in CORnet-S than in CORnet-Z, demonstrating that recurrent and deep feedforward DNNs better predict location representations in LOC than shallow DNNs. **e,** Classification of horizontal locations (left vs. right) across categories in the high clutter condition (analogous to the cross-hemifield classification of ROI data, Fig. 4a). **f,** Classification of vertical locations (up vs. down) across categories in the high clutter condition (analogous to the within-hemifield classification of ROI data, Fig. 4b). Both horizontal and vertical location classification yielded results that were qualitatively similar to the main analysis. Differences between horizontal and vertical location classification were small and showed no clear pattern: when subtracting vertical from horizontal classification accuracies at the top layers of each DNN, we found slightly higher accuracies in the vertical classification for CORnet-S and ResNet-50 (difference CORnet-S: -1.86%; difference ResNet-50: -1.43%) and slightly higher accuracies in the horizontal classification for Alexnet and CORnet-Z (difference Alexnet: 0.16%; difference CORnet-Z: 1.09%).

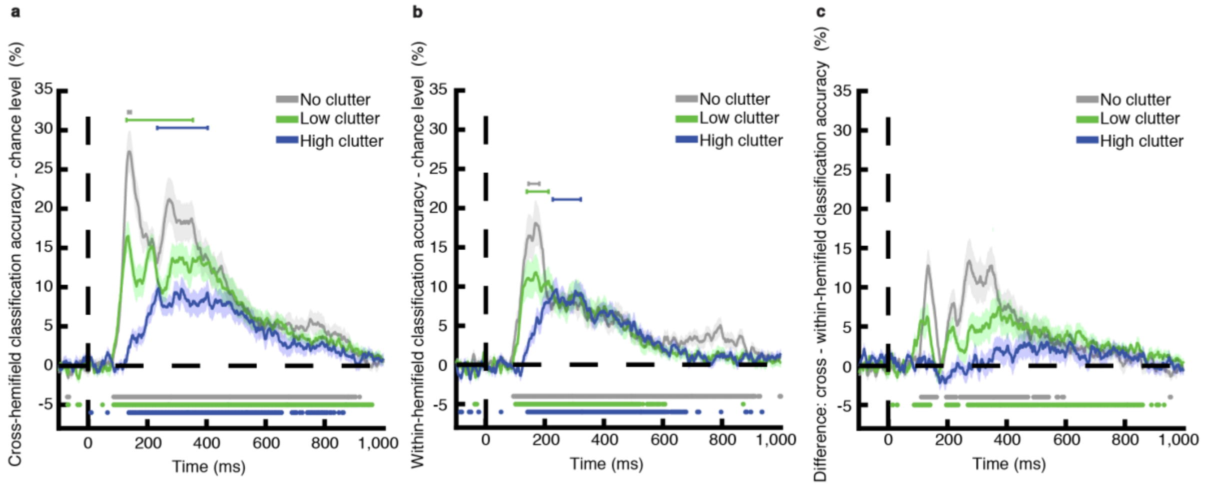

Supplementary Figure 4. Category-independent location classification across and within visual hemifields. **a,** Results of time-resolved cross-hemifield (left vs. right) location classification across category from EEG data (N=27, two-tailed Wilcoxon signed-rank test, P<0.05 FDR corrected). Conventions as in Fig. 5a. Cross-hemifield location information peaked at 137 ms (135–144 ms), 135 ms (129–354 ms) and 237 ms (233-404 ms) in the no, low and high clutter conditions, respectively. The peak in the high clutter condition was significantly later than the peaks in the no (100 ms (91–266 ms); P<0.001) and the low clutter conditions (102 ms (17–270 ms), P=0.032; N=27, bootstrap test, 10,000 bootstraps, FDR corrected). There was no significant delay between the no and low clutter conditions (2 ms (-11–196 ms), P=0.34). **b,** Results of time-resolved within-hemifield (up vs. down) location classification across category from EEG data (N=27, two-tailed Wilcoxon signed-rank test, P<0.05 FDR corrected). Conventions as in Fig. 5a. Within-hemifield location information peaked at 168 ms (145–182 ms), 171 ms (139–213 ms) and 240 ms (227-322 ms) in the no, low and high clutter conditions, respectively. Like in previous analyses, the peak in the high clutter condition was significantly delayed compared to the peaks in the no (72 ms (55–153 ms); P<0.001) and the low clutter conditions (69 ms (31–165 ms), P=0.003). Consistent with all other analyses, the delay between the no and low clutter conditions did not reach significance (3 ms (-35–40 ms), P=0.64; N=27, bootstrap test, 10,000 bootstraps, FDR corrected). **c,** Difference curves of across minus within-hemifield location classification. Location information was significantly higher in the cross-hemifield than in the within-hemifield classification in the no and low clutter conditions, but not in the high clutter condition (N=27, two-tailed Wilcoxon signed-rank test, P<0.05 FDR corrected).

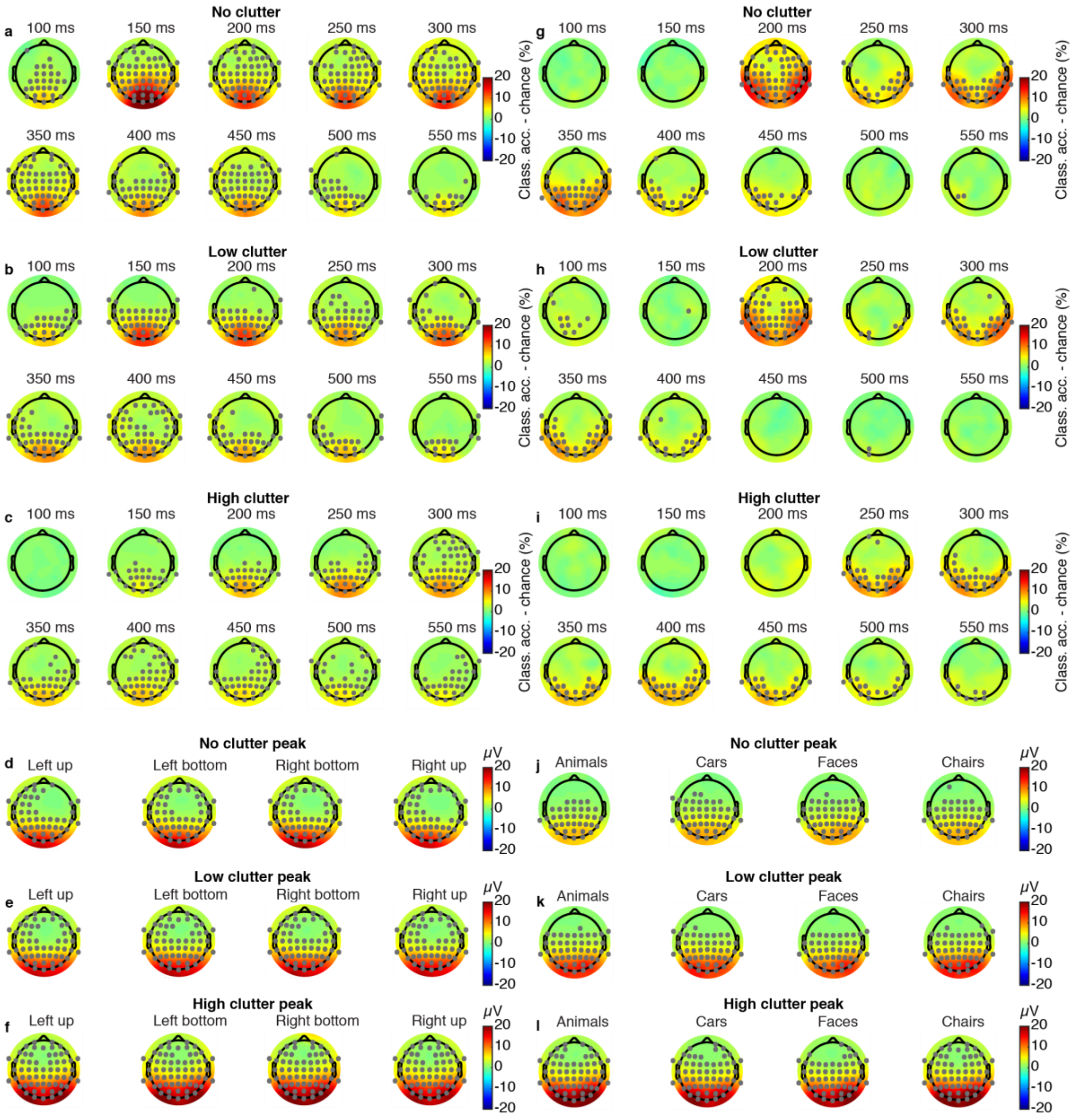

Supplementary Figure 5. Univariate and multivariate EEG topographies. Significant electrodes are marked in grey (*N*=27, two-sided Wilcoxon signed-rank test, *P*<0.05, FDR corrected across electrodes and time points). **a**, **b**, **c**, Location classification across categories in the no (**a**), low (**b**) and high clutter (**c**) conditions across time in 50 ms time steps. **d**, **e**, **f**, Univariate EEG topographies at the peaks in the no (**d**), low (**e**) and high clutter (**f**) conditions. Locations were averaged across categories. **g**, **h**, **i**, Category classification across locations in the no (**g**), low (**h**) and high clutter (**i**) conditions across time. **j**, **k**, **l**, Univariate EEG topographies at the peaks in the no (**j**), low (**k**) and high clutter (**l**) conditions. Categories were averaged across locations.

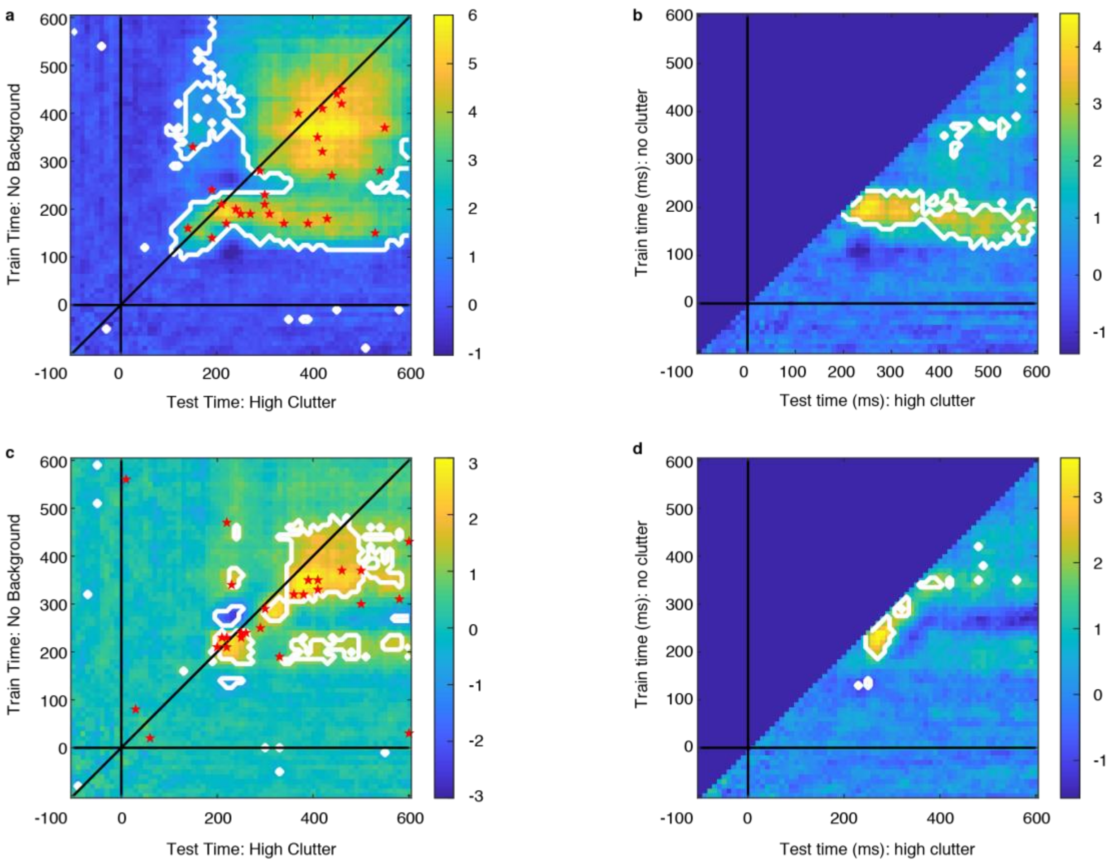

Supplementary Figure 6. Quantification of asymmetry with respect to the diagonal in time-generalization matrices. a, Single subject peaks of the result in Fig. 5e (temporal generalization of location across categories and backgrounds) are marked as red stars. The group-averaged peak of the cluster in Fig. 5e was significantly shifted below the diagonal (Euclidean distance=49.50 ms; 10,000 bootstraps; P=0.01; 95% CI=14.14–77.78). **b,** Result of subtracting the upper- from the lower diagonal of the result in Fig. 5e (temporal generalization of location across categories and backgrounds). Significant time points are outlined in white (N=27, two-tailed Wilcoxon signed-rank test, P<0.05 FDR corrected). **c**, Single subject peaks of the result in Fig. 6g (temporal generalization of category across locations and backgrounds) are marked as red stars. The group-averaged peak was not significantly shifted below the diagonal (Euclidean distance=28.28 ms; 10,000 bootstraps; P=0.23; 95% CI= -7.07–35.35). **d**, Result of subtracting the upper- from the lower diagonal of the result in Fig. 6g (temporal generalization of category across locations and backgrounds). Significant time points are outlined in white (N=27, two-tailed Wilcoxon signed-rank test, P<0.05 FDR corrected).

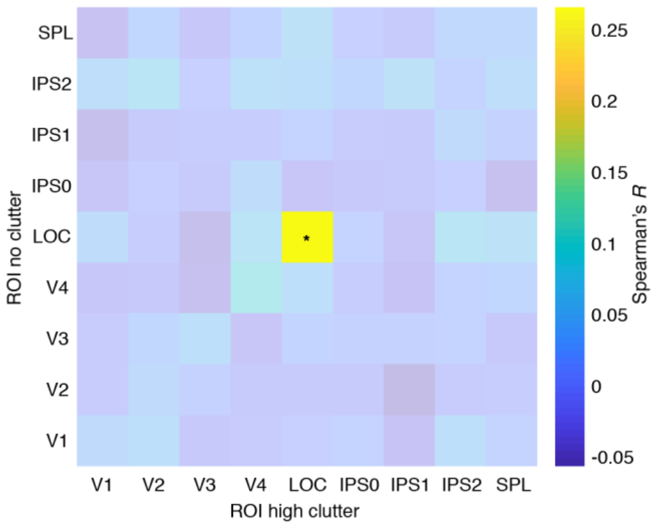

Supplementary Figure 7. Results of RSA comparing location representations across ROIs and backgrounds. To investigate whether regions of early visual, the dorsal or the ventral visual stream shared location representations tolerant to category across the no and high clutter conditions, we performed representational similarity analysis [1,2], comparing representations in the no clutter and high clutter conditions between and within ROIs. For each ROI and background, an RDM contained the pairwise classification accuracies between all pairs of locations (6) and all training and testing pairs across categories (6) in both training and testing directions (2), resulting in a 72 × 1 vector. For each participant, we computed the Spearman's Rho correlation across nine ROI RDMs and across the no clutter and high clutter condition, resulting in a 9 × 9 matrix of correlations between the two background conditions. The resulting matrices were then averaged across participants. The representations within LOC were significantly correlated across background conditions (indicated by a star), but not within or across other ROIs (N=25, two-tailed Wilcoxon-signed-rank test, P<0.05, FDR-corrected).

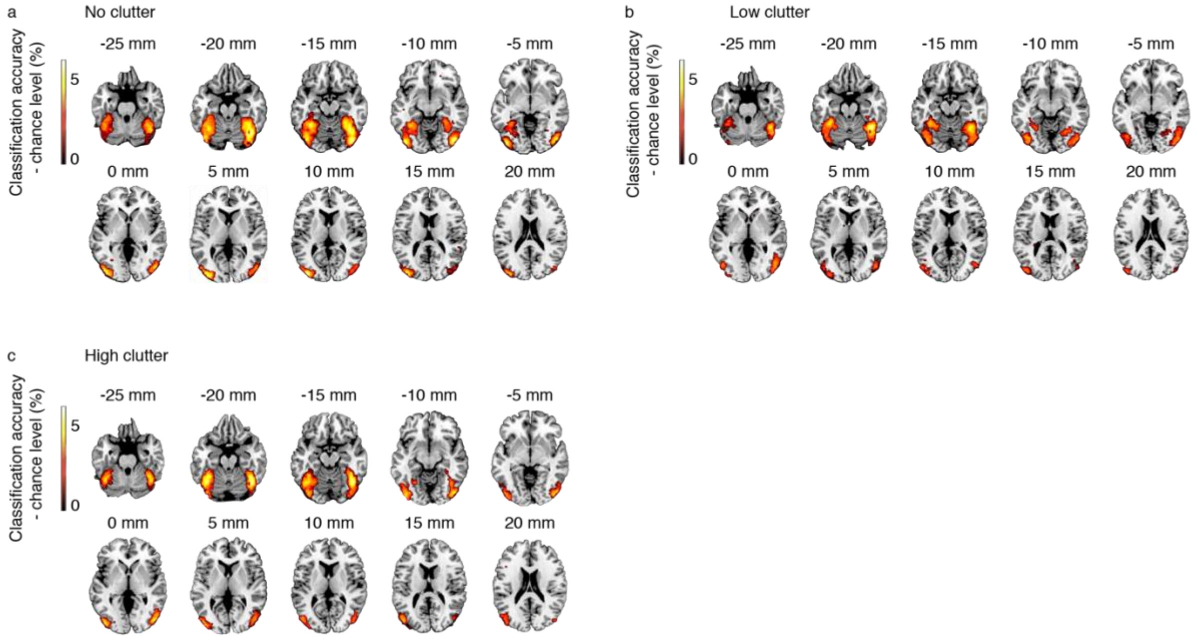

Supplementary Figure 8. Axial slices for searchlight category classification across location in the a, no clutter b, low clutter and c, high clutter conditions. Colored areas indicate significant category classification across location within that background condition (*N*=25, cluster-permutation test with threshold-free cluster enhancement (TFCE[3]; as implemented in CoSMoMVPA[4]. TFCE is a cluster definition method that is independent of an arbitrarily defined cluster-threshold by the experimenter), 10,000 permutations, *P*=0.017, Bonferroni corrected for 3 background conditions). Millimeters (mm) indicate axial slice position along z-axis in MNI space.

Supplementary Tables

Analysis	ROI	*P*-values		
		**No clutter**	**Low clutter**	**High clutter**
**Location**				
	V1	<.001	<.001	<.001
	V2	<.001	<.001	.004
	V3	<.001	<.001	<.001
	V4	<.001	<.001	<.001
	LOC	<.001	<.001	<.001
	IPS0	.014	.139	.028
	IPS1	.004	.150	.211
	IPS2	<.001	.002	.033
	SPL	<.001	.001	.004
	IPS3	0.046	0.085	0.079
	IPS4	0.276	0.706	0.989
	IPS5	0.946	0.797	0.638
**Category**				
	V1	.112	.153	.021
	V2	.700	.281	.252
	V3	.106	.052	.045
	V4	<.001	<.001	<.001
	LOC	<.001	<.001	<.001
	IPS0	.264	.914	.005
	IPS1	.459	.412	.397
	IPS2	.914	.511	.545
	SPL	.777	.063	.679

Supplementary Table 1. Uncorrected *P*-values from the two-sided Wilcoxon signed-rank test, testing a) location (Fig. 3b) and b) category (Fig. 6b) classification accuracy vs. chance level (50%) in early visual, ventral and dorsal ROIs. On these *P*-values, we applied an FDR correction for all 15 values (9 ROIs × 3 background conditions).

Post-hoc comparison within background			Statistical Parameters				
**Background**	**ROI**	**ROI**	**Mean difference**	**SEM**	**Degrees of freedom**	***t*-value**	**Tukey-corrected *P*-value**
**No Clutter**							
	V1	V2	4.73	1.50	24	3.15	0.17
		V3	-3.28	1.74	24	-1.88	0.84
		V4	-5.54	1.53	24	-3.12	0.07
		LOC	-2.37	1.83	24	-1.29	0.99
	V2	V3	-8.00	1.78	24	-4.50	0.01
		V4	-10.27	1.89	24	-5.43	0.001
		LOC	-7.10	1.96	24	-3.63	0.07
	V3	V4	-2.27	2.09	24	-1.08	1.00
		LOC	0.91	2.03	24	0.45	1.00
	V4	LOC	3.17	1.59	24	2.00	0.78
**Low Clutter**							
	V1	V2	2.78	1.18	24	2.36	0.56
		V3	-2.40	1.54	24	-1.56	0.95
		V4	-5.70	1.45	24	-3.94	0.03
		LOC	-8.00	1.90	24	-4.21	0.02
	V2	V3	-5.18	1.21	24	-4.27	0.02
		V4	-8.48	1.26	24	-6.75	<.001
		LOC	-10.78	1.37	24	-7.88	<.001
	V3	V4	-3.30	1.42	24	-2.33	0.58
		LOC	-5.60	1.15	24	-4.89	0.004
	V4	LOC	-2.30	1.41	24	-1.64	0.93
**High Clutter**							
	V1	V2	1.52	1.27	24	1.20	1.00
		V3	-1.72	1.47	24	-1.17	1.00
		V4	-4.53	1.55	24	-2.92	0.25
		LOC	-8.98	2.06	24	-4.36	0.01
	V2	V3	-3.24	1.12	24	-2.89	0.27
		V4	-6.06	1.38	24	-4.40	0.01
		LOC	-10.51	1.51	24	-6.95	<.001
	V3	V4	-2.82	1.25	24	-2.25	0.63
		LOC	-7.27	1.22	24	-6.00	<.001
	V4	LOC	-4.45	1.30	24	-3.42	0.10

Supplementary Table 2. Post-hoc tests comparing ventral ROIs within background condition, following up on significant ventral ROI × background interaction for the classification of location across category (fMRI experiment, Fig. 3b). In sum, location information was significantly lower in early visual regions V1, V2 and V3 than in LOC in the low and high clutter conditions. In the no clutter condition, location information was lower in region V2 than in region V3 and V4.

Post-hoc comparison within background			Statistical Parameters				
**Background**	**ROI**	**ROI**	**Mean difference**	**SEM**	**Degrees of freedom**	***t*-value**	**Tukey-corrected *P*-value**
**No Clutter**							
	V1	V2	4.73	1.50	24	3.15	0.25
		V3	-3.28	1.74	24	-1.88	0.93
		IPS0	13.61	1.53	24	8.92	<.001
		IPS1	13.75	1.48	24	9.31	<.001
		IPS2	11.72	1.45	24	8.10	<.001
		SPL	10.49	1.41	24	7.43	<.001
	V2	V3	-8.01	1.78	24	-4.50	0.02
		IPS0	8.88	1.69	24	5.26	0.003
		IPS1	9.02	1.45	24	6.22	<.001
		IPS2	6.99	1.53	24	4.57	0.01
		SPL	5.77	1.54	24	3.75	0.08
	V3	IPS0	16.88	2.15	24	7.84	<.001
		IPS1	17.03	1.96	24	8.70	<.001
		IPS2	14.99	1.79	24	8.39	<.001
		SPL	13.77	2.03	24	6.80	<.001
	IPS0	IPS1	0.14	0.71	24	0.20	1.00
		IPS2	01.89	.85	24	-2.22	0.78
		SPL	-3.11	0.71	24	-4.39	0.02
	IPS1	IPS2	-2.03	0.83	24	-2.46	0.64
		SPL	-3.26	0.80	24	-4.06	0.04
	IPS2	SPL	-1.22	0.79	24	-1.55	0.99
**Low Clutter**							
	V1	V2	2.78	1.78	24	2.36	0.70
		V3	-2.40	1.54	24	-1.56	0.99
		IPS0	7.84	1.31	24	5.97	<.001
		IPS1	7.84	1.41	24	5.57	0.001
		IPS2	5.71	1.57	24	3.65	0.10
		SPL	5.66	1.58	24	3.58	0.12
	V2	V3	-5.18	1.21	24	-4.27	0.03
		IPS0	5.06	0.93	24	5.42	0.002
		IPS1	5.06	1.02	24	4.94	0.006
		IPS2	2.93	1.05	24	2.78	0.44
		SPL	2.87	1.24	24	2.32	0.72
	V3	IPS0	10.24	1.26	24	8.12	<.001
		IPS1	10.24	1.26	24	8.11	<.001
		IPS2	8.11	1.12	24	7.22	<.001
		SPL	8.06	1.32	24	6.12	<.001
	IPS0	IPS1	0.00	0.70	24	0.00	1.00
		IPS2	-2.13	0.82	24	2.60	0.55
		SPL	-2.18	0.85	24	-2.57	0.57
	IPS1	IPS2	-2.13	0.81	24	-2.62	0.54
		SPL	-2.18	0.71	24	-3.07	0.29
	IPS2	SPL	-0.06	0.68	24	-0.08	1.00

**High Clutter**							
	V1	V2	1.52	1.27	24	1.20	1.00
		V3	-1.72	1.47	24	-1.17	1.00
		IPS0	2.85	1.54	24	1.85	0.94
		IPS1	3.75	1.45	24	2.58	0.56
		IPS2	2.65	1.38	24	1.93	0.91
		SPL	1.66	1.62	24	1.02	1.00
	V2	V3	-3.24	1.12	24	-2.89	0.38
		IPS0	1.33	0.96	24	1.39	1.00
		IPS1	2.23	0.92	24	2.42	0.67
		IPS2	1.13	0.84	24	1.34	1.00
		SPL	0.13	0.85	24	0.16	1.00
	V3	IPS0	4.57	1.09	24	4.20	0.03
		IPS1	5.47	1.23	24	4.46	0.02
		IPS2	4.37	0.91	24	4.78	0.008
		SPL	3.37	0.98	24	3.44	0.15
	IPS0	IPS1	0.90	0.64	24	1.41	1.00
		IPS2	-0.20	0.89	24	-0.23	1.00
		SPL	-1.19	0.76	24	-1.58	0.98
	IPS1	IPS2	-1.10	0.75	24	-1.48	0.99
		SPL	-2.09	0.86	24	-2.44	0.65
	IPS2	SPL	-0.99	0.86	24	-1.16	1.00

Supplementary Table 3. Post-hoc tests comparing dorsal ROIs within background condition, following up on significant dorsal ROI × background interaction for the classification of location across category (fMRI experiment, Fig. 3b). In sum, location information was significantly higher in early visual regions V1, V2 and V3 than in dorsal regions in the no and low clutter conditions. In the high clutter condition, location information was higher in V3 than in IPS0, IPS1 and IPS2.

**Post-hoc comparison within background**			**Statistical Parameters**				
**Background**	**ROI**	**ROI**	**Mean difference**	**SEM**	**Degrees of freedom**	***t*-value**	**Tukey-corrected *P*-value**
**No Clutter**							
	V1	V3	0.07	0.01	24	5.63	0.002
		V4	0.06	0.01	24	4.67	0.02
		LOC	0.06	0.01	24	5.53	0.002
		IPS0	0.07	0.01	24	6.75	<.001
		IPS1	0.07	0.01	24	6.12	<.001
		IPS2	0.05	0.01	24	4.55	0.02
	V2	V3	0.04	0.01	24	4.67	0.02
		IPS0	0.04	0.01	24	5.08	0.006
**Low Clutter**							
	V2	V3	0.04	0.01	24	5.14	0.006
	V3	IPS2	-0.05	0.01	24	-4.32	0.04
		SPL	-0.09	0.02	24	-4.83	0.01
**High Clutter**							
	V1	V3	0.05	0.01	24	5.13	0.006
	V2	V3	0.04	0.01	24	5.15	0.005
	V3	SPL	-0.10	0.02	24	4.19	0.05

Supplementary Table 4. Significant post-hoc tests comparing ROIs within background condition, following up on significant ROI × background interaction for absolute t-values averaged across location and background conditions.

Analysis	Background	Peak classification value - chance level (%)	Significance onset (ms)	Peak latency (ms) (CI (ms))
**Location**				
	No Clutter	23	86	140 (133–147)
	Low Clutter	14	88	133 (121–233)
	High Clutter	10	134	317 (250–336)
**Category**				
	No Clutter	12	171	215 (213–219)
	Low Clutter	8	178	215 (203–236)
	High Clutter	5	205	233 (214–303)

Supplementary Table 5. Classification peak values, significance onset and peak latencies for the time-resolved classification in the EEG experiment. Confidence intervals are given in brackets. Classification onsets and peaks emerged later in the high clutter condition, both for the classification of location (Fig. 5a) and category (Fig. 6c). Significance was determined using a bootstrap test on peak-to-peak latency differences between background conditions (see Methods).

Post-hoc comparison within background			Statistical Parameters				
**Background**	**ROI**	**ROI**	**Mean difference**	**SEM**	**Degrees of freedom**	***t*-value**	**Tukey-corrected *P*-value**
**No Clutter**							
	V1	V4	-0.30	0.04	24	-7.10	<.001
		LOC	-0.33	0.06	24	-5.64	<.001
	V2	V4	-0.23	0.04	24	-6.21	<.001
		LOC	-0.25	0.06	24	-4.37	0.006
	V3	V4	-0.19	0.04	24	-5.06	0.001
		LOC	-0.22	0.06	24	-3.87	0.021

Supplementary Table 6. Significant post-hoc tests comparing ROIs within background condition, following up on significant ROI × background interaction for EEG-fMRI fusion in Fig. 5f.

Post-hoc comparison		Statistical Parameters				
**ROI**	**ROI**	**Mean difference**	**SEM**	**Degrees of freedom**	***t*-value**	**Tukey-corrected *P*-value**
**V1**						
	V2	-0.18	0.32	24	-0.57	0.98
	V3	-1.03	0.48	24	-2.16	0.23
	V4	-3.85	0.82	24	-4.68	<.001
	LOC	-4.19	0.61	24	-6.84	<.001
**V2**						
	V3	-0.85	0.53	24	-1.59	0.52
	V4	-3.67	0.79	24	-4.63	<.001
	LOC	-4.01	0.62	24	-6.49	<.001
**V3**						
	V4	-2.82	0.56	24	-5.06	<.001
	LOC	-3.17	0.46	24	-6.84	<.001
**V4**						
	LOC	-0.34	0.73	24	-0.48	0.99

Supplementary Table 7. Post-hoc tests following up on the significant main effect of ventral ROI for category classification across location in the fMRI experiment (Fig. 6b).

Cluster	Background	Significance onset (ms)	Peak latency (ms)
**1st cluster**			
	No Clutter	180	220
	High Clutter	190	220
**2nd cluster**			
	No Clutter	270	280
	High Clutter	300	320
**3rd cluster**			
	No Clutter	310	360
	High Clutter	350	400

Supplementary Table 8. Timing details for the result of the time-generalization analysis of category across location and background in Fig. 6g.

Supplementary References

1. Kriegeskorte, N., Mur, M. & Bandettini, P. Representational similarity analysis - connecting the branches of systems neuroscience. *Front. Syst. Neurosci.* **2**, 4 (2008).
2. Kriegeskorte, N. & Kievit, R. A. Representational geometry: Integrating cognition, computation, and the brain. *Trends Cogn. Sci.* **17**, 401–412 (2013).
3. Smith, S. M. & Nichols, T. E. Threshold-free cluster enhancement: Addressing problems of smoothing, threshold dependence and localisation in cluster inference. *Neuroimage* **44**, 83–98 (2009).
4. Oosterhof, N. N., Connolly, A. C. & Haxby, J. V. CoSMoMVPA: Multi-modal multivariate pattern analysis of neuroimaging data in Matlab/GNU Octave. *Front. Neuroinform.* **10**, 1–27 (2016).
