# Supplementary material for: The spatiotemporal neural dynamics of object location representations in the human brain
Source: Nat Hum Behav. 2022 Feb 24;6(6):796–811. doi: 10.1038/s41562-022-01302-0 (PMC9225954; doi:10.1038/s41562-022-01302-0)
Supplement: Supplementary file 2 — Reporting summary [file 41562_2022_1302_MOESM2_ESM.pdf]

nature research

Corresponding author(s): Monika Graumann
Radoslaw Martin Cichy

Last updated by author(s): Dec 17, 2021

Reporting Summary

Nature Research wishes to improve the reproducibility of the work that we publish. This form provides structure for consistency and transparency in reporting. For further information on Nature Research policies, see our Editorial Policies and the Editorial Policy Checklist.

Statistics

For all statistical analyses, confirm that the following items are present in the figure legend, table legend, main text, or Methods section.

n/a	Confirmed	
☐	☒	The exact sample size (n) for each experimental group/condition, given as a discrete number and unit of measurement
☐	☒	A statement on whether measurements were taken from distinct samples or whether the same sample was measured repeatedly
☐	☒	The statistical test(s) used AND whether they are one- or two-sided<br>*Only common tests should be described solely by name; describe more complex techniques in the Methods section.*
☒	☐	A description of all covariates tested
☐	☒	A description of any assumptions or corrections, such as tests of normality and adjustment for multiple comparisons
☐	☒	A full description of the statistical parameters including central tendency (e.g. means) or other basic estimates (e.g. regression coefficient) AND variation (e.g. standard deviation) or associated estimates of uncertainty (e.g. confidence intervals)
☐	☒	For null hypothesis testing, the test statistic (e.g. F, t, r) with confidence intervals, effect sizes, degrees of freedom and P value noted<br>*Give P values as exact values whenever suitable.*
☒	☐	For Bayesian analysis, information on the choice of priors and Markov chain Monte Carlo settings
☒	☐	For hierarchical and complex designs, identification of the appropriate level for tests and full reporting of outcomes
☐	☒	Estimates of effect sizes (e.g. Cohen's d, Pearson's r), indicating how they were calculated

Our web collection on statistics for biologists contains articles on many of the points above.

Software and code

Policy information about availability of computer code

Data collection	The data was collected using Matlab and the experimental paradigms were presented using the Psychophysics Toolbox Version 3.0.12 (PTB-3).
Data analysis	For the data preprocessing and analysis we used the following software: MATLAB R2018b, EEGLAB toolbox (version 14), SASICA plugin for EEGLAB, LIBSVM-3.11, SPM8 toolbox, CoSMoMVPA toolbox.

For manuscripts utilizing custom algorithms or software that are central to the research but not yet described in published literature, software must be made available to editors and reviewers. We strongly encourage code deposition in a community repository (e.g. GitHub). See the Nature Research guidelines for submitting code & software for further information.

Data

Policy information about availability of data

All manuscripts must include a data availability statement. This statement should provide the following information, where applicable:

- Accession codes, unique identifiers, or web links for publicly available datasets
- A list of figures that have associated raw data
- A description of any restrictions on data availability

The experimental stimuli used in this study, the fMRI and EEG data as well as neural network activations are publicly available via https://osf.io/7zswn/?view_only=21a714db58584ffeb2837fc0548bf659.

Field-specific reporting

Please select the one below that is the best fit for your research. If you are not sure, read the appropriate sections before making your selection.

☐ Life sciences ☒ Behavioural & social sciences ☐ Ecological, evolutionary & environmental sciences

For a reference copy of the document with all sections, see nature.com/documents/nr-reporting-summary-flat.pdf

Behavioural & social sciences study design

All studies must disclose on these points even when the disclosure is negative.

Study description	In this study we recorded quantitative data separately from two experiments. 1) 3 Tesla functional magnetic resonance imaging (fMRI) data to acquire human brain activity data with high spatial resolution. 2) Electroencephalography (EEG) data to acquire human brain activity data with high temporal resolution. In both experiments, participants were performing a visual task while we recorded data.
Research sample	29 participants participated in the EEG experiment of which two were excluded because of equipment failure (N=27, mean age 26.8 years, SD=4.3, 22 female). 25 participants (mean age 28.8, SD=4.0, 17 female) completed the fMRI experiment. The participant pools of the experiments did not overlap except for two participants. All participants provided informed consent prior to the studies and received a monetary reward or course credit for their participation.
Sampling strategy	Participants were selected according to the following requirements: 18-40 years old, with normal or corrected-to-normal vision, fulfillment of the MR security criteria (no implants or metal parts, tattoos, non-removable piercing, claustrophobia, pregnancy, neurological disorders, etc.).<br>Sample size was chosen to exceed comparable M/EEG and fMRI classification studies to enhance power.
Data collection	During both experiments, participants' responses were recorded with a computer, while the ongoing brain activity during the task was recorded using the 3T fMRI scanner (experiment 1) and the EEG (experiment 2). No one was present in the room together with the participants during the experiments. Blinding to the experimental conditions or the study hypothesis was not possible, but data was analyzed using a single pipeline for all subjects.
Timing	1) fMRI experiment: the data collection started February 2019 and ended in March 2019. 2) EEG experiment: the data collection started in May 2017 and ended in November 2017, with a short gap from July to September 2017 for data analysis.
Data exclusions	1) No participants were excluded in the fMRI experiment. 2) Two participants were excluded in the EEG experiment because of equipment failure.
Non-participation	No participants declined participation or dropped out.
Randomization	Participants were not allocated into experimental groups.

Reporting for specific materials, systems and methods

We require information from authors about some types of materials, experimental systems and methods used in many studies. Here, indicate whether each material, system or method listed is relevant to your study. If you are not sure if a list item applies to your research, read the appropriate section before selecting a response.

Materials & experimental systems

n/a	Involved in the study	
☒	☐	Antibodies
☒	☐	Eukaryotic cell lines
☒	☐	Palaeontology and archaeology
☒	☐	Animals and other organisms
☐	☒	Human research participants
☒	☐	Clinical data
☒	☐	Dual use research of concern

Methods

n/a	Involved in the study	
☒	☐	ChIP-seq
☒	☐	Flow cytometry
☐	☒	MRI-based neuroimaging

Human research participants

Policy information about studies involving human research participants

Population characteristics	See above.
Recruitment	Participants were recruited using the mailing lists for study participation of the psychology program, of the cognitive

Recruitment | neuroscience program and of the medical studies program from the following Berlin universities: Freie Universität Berlin, Humboldt Universität zu Berlin, Charite.

Ethics oversight | The study was approved by the ethics committee of the Department of Education and Psychology of the Freie Universität Berlin, Germany.

Note that full information on the approval of the study protocol must also be provided in the manuscript.

Magnetic resonance imaging

Experimental design

Design type: Event-related fMRI design.

Design specifications: Each participant completed one fMRI recording session consisting of 10 runs (run duration: 552 s), resulting in 92 minutes of fMRI recording of the main experiment. During each run, each of the 144 images of the stimulus set was shown once (regular trials). Image duration was 0.5 s, with a 2.5 s inter-stimulus-interval (ISI). Regular trials were interspersed every 3rd to 5th trial (equally probable, in total 36 per run) with catch trials. Catch trials repeated the image shown on the previous trial. Participants were instructed to respond with a button press to these repetitions (i.e. a one-back task).

Behavioral performance measures: Button presses and response times were recorded for each subject during the experiment. Responses were recorded to ensure that participants were directing their attention towards the stimuli. Response trials were excluded from analysis.

Acquisition

Imaging type(s): functional and structural MRI

Field strength: 3 Tesla

Sequence & imaging parameters: We acquired functional images covering the entire brain using a T2*-weighted gradient-echo planar sequence (TR=2, TE=30 ms, 70° flip angle, 3-mm3 voxel size, 37 slices, 20% gap, 192-mm field of view, 64 × 64 matrix size, interleaved acquisition).

Area of acquisition: Whole brain.

Diffusion MRI ☐ Used ☒ Not used

Preprocessing

Preprocessing software: We preprocessed fMRI data using SPM8. This involved realignment, coregistration and normalization to the structural MNI template brain. FMRI data from the localizer was smoothed with an 8 mm FWHM Gaussian kernel, but the main experiment data was left unsmoothed.

Normalization: The normalization method applied on all functional brain data was non-linear. We entered the subject specific T1 structural image as source image and the MNI standard T1 provided in the SPM8 toolbox as template image.

Normalization template: We used the T1 template in MNI space provided in the SPM8 toolbox.

Noise and artifact removal: To remove movement artifacts from the fMRI time-series, we realigned the functional brain images in SPM8 using default parameters. In the GLM, movement parameters were entered as nuisance regressors. We applied no artifact removal for heart rate and respiration.

Volume censoring: Was not applied.

Statistical modeling & inference

Model type and settings: We performed multivariate pattern analysis on the brain activity data. Specifically, we trained and tested support-vector machines on the individual participants' data and performed a statistical analysis on classification results.

Effect(s) tested: Whole-brain: for all voxels, we tested whether classification accuracies significantly exceeded chance level. This was done separately for three background conditions (no, low and high background clutter).
ROI: using a repeated-measures ANOVA with a 5×3 design, we tested for the interaction between 5 regions-of-interest in the ventral stream (V1, V2, V3, V4, LOC) and 3 background conditions (no, low and high cluttered backgrounds).
Another repeated measures ANOVA with 7 ×3 design tested the interaction between 7 regions-of-interest in the dorsal stream (V1,V2,V3,IPS0,IPS1,IPS2,SPL) and 3 background conditions (no, low and high cluttered backgrounds).
When the assumption of sphericity was violated, the degrees of freedom were corrected using the Greenhouse-Geisser estimates of sphericity.

Specify type of analysis: ☐ Whole brain ☐ ROI-based ☒ Both

Anatomical location(s)

We first defined ROIs in early visual cortex (V1, V2, V3), in the ventral stream (V4, LOC) and in the dorsal stream (IPS0,IPS1,IPS2,SPL) using anatomical masks from a probabilistic atlas (Wang et al., 2015) for both hemispheres combined. To avoid overlap between the ROI masks we removed all overlapping voxels. In a second step we selected the 325 most activated voxels in the participant-specific localizer results, using the objects > scrambled contrast for LOC and the objects & scrambled objects > baseline contrast for the remaining ROIs. This yielded participant-specific ROI definitions.

Statistic type for inference
(See Eklund et al. 2016)

We tested whether classification accuracies significantly exceeded chance-level. This was done per ROI and in the the whole-brain searchlight it was done voxel-wise. In both cases we tested this with non-parametric, two-tailed Wilcoxon signed rank tests. In each case the null hypothesis was that the observed classification accuracies came from a distribution with a median of chance level performance (i.e., 50% for pairwise classification).

Correction

The P-values resulting from the Wilcoxon signed rank tests were corrected for multiple comparisons using false discovery rate at 5% level under the assumption of independent or positively correlated tests.

Models & analysis

n/a	Involved in the study
☒	☐ Functional and/or effective connectivity
☒	☐ Graph analysis
☐	☒ Multivariate modeling or predictive analysis

Multivariate modeling and predictive analysis

For the ROI-based analysis, for each ROI separately we extracted and arranged t-values into pattern vectors for each of the 48 conditions and 10 runs. To increase the SNR, we randomly binned run-wise pattern vectors into five bins of two runs which were averaged, resulting in five pseudo-run pattern vectors. We then performed 5-fold leave-one-pseudo-run-out-cross validation. In detail, we assigned four pseudo-trials per location condition of the same category to the training set. We then tested the SVM on one pseudo-trial for each of the same two location conditions, but now from a different category yielding percent classification accuracy (50% chance level) as output. Equivalent SVM training and testing was repeated for all combinations of location and category pairs before results were averaged. The result reflects how much category-tolerant location information was present for each ROI, participant and background condition separately.

The searchlight procedure was conceptually equivalent to the ROI-based analysis with the difference of the selection of voxel patterns entering the analysis.
